# Supplementary material for: Homozygous MTAP deletion in primary human glioblastoma is not associated with elevation of methylthioadenosine
Source: Nat Commun. 2021 Jul 9;12:4228. doi: 10.1038/s41467-021-24240-3 (PMC8270912; doi:10.1038/s41467-021-24240-3)
Supplement: Supplementary file 1 — Supplementary Information [file 41467_2021_24240_MOESM1_ESM.pdf]

Supplementary Information

Homozygous *MTAP* deletion in primary human glioblastoma is not associated with elevation of methylthioadenosine

Yasaman Barekatain[1,2,3]*, Jeffrey J. Ackroyd[1,3], Victoria C. Yan[1,3], Sunada Khadka[1,2,3], Lin Wang[4], Ko-Chien Chen[2,3], Anton H. Poral[1], Theresa Tran[1], Dimitra K. Georgiou[1], Kenisha Arthur[1], Yu-Hsi Lin[1], Nikunj Satani[1], Elliot S. Ballato[1], Eliot Behr[1,2], Ana deCarvalho[5], Roel Verhaak[6], John de Groot[7], Jason T. Huse[8], John M. Asara[9], Raghu Kalluri[2], Florian L. Muller[1,10]*

Supplementary Figure 1: Genomic deletion of *MTAP* as part of the 9p21 locus and the associated metabolic vulnerabilities.

Supplementary Figure 2: Measurements of MTA levels of *MTAP*-intact versus deleted cancer cell lines vary depending on how well intracellular versus secreted metabolites are separated during sample preparation.

Supplementary Figure 3: *MTAP* status of the panel of cell lines used in this study is confirmed by immunoblotting.

Supplementary Figure 4: Intracellular SAM as a normalization factor for MTA across different cell culture experiments.

Supplementary Figure 5: No significant elevation of MTA in *MTAP*-deleted human GBM tumors (MDA series).

Supplementary Figure 6: No correlation between *MTAP* expression and MTA levels in primary human GBM tumors.

Supplementary Figure 7: Minimal and insignificant elevation of MTA in primary GBM tumors compared to low-grade gliomas.

Supplementary Figure 8: SDMA levels are less in *MTAP*-deleted cells compared to the *MTAP*-intact cells.

Supplementary Figure 9: The effects of culturing cells in the extremely low methionine and cysteine condition on MTA levels.

Supplementary Figure 10: Comparison between intracellular and extracellular MTA levels in cells cultured in DMEM versus Plasmax.

Supplementary Figure 11: Not significant elevation of MTA in cerebrospinal fluid (CSF) and venous plasma collected from GBM.

Supplementary Figure 12: Validation of an MTAP rabbit monoclonal antibody to detect *MTAP*-deleted tumors by IHC on FFPE sections.

Supplementary Figure 13: IHC Staining with the MTAP monoclonal antibody remains specific even at longer exposures.

Supplementary Figure 14: MTAP IHC developed by EnzMet with or without Eosin counterstain yields increased histological resolution.

Supplementary Figure 15: Lower stromal content in human xenograft in mice compared to human primary GBM tumors.

Supplementary Figure 16: Strong correlation between residual MTAP staining and microglia content in *MTAP*-deleted human GBM tumors.

Supplementary Figure 17: Representative GBM tumors stained with anti-MTAP antibody differing in stromal content.

Supplementary Figure 18: The co-culture of *MTAP*-deleted with *MTAP*-intact cells abrogates MTA accumulation in conditioned media.

Supplementary Figure 19: Exogenous MTA is consumed by *MTAP*-intact cells through the methionine salvage pathway.

Supplementary Figure 20: The co-culture of *MTAP*-deleted and *MTAP*-rescued glioma cells abrogates MTA accumulation from conditioned media.

Supplementary Figure 1

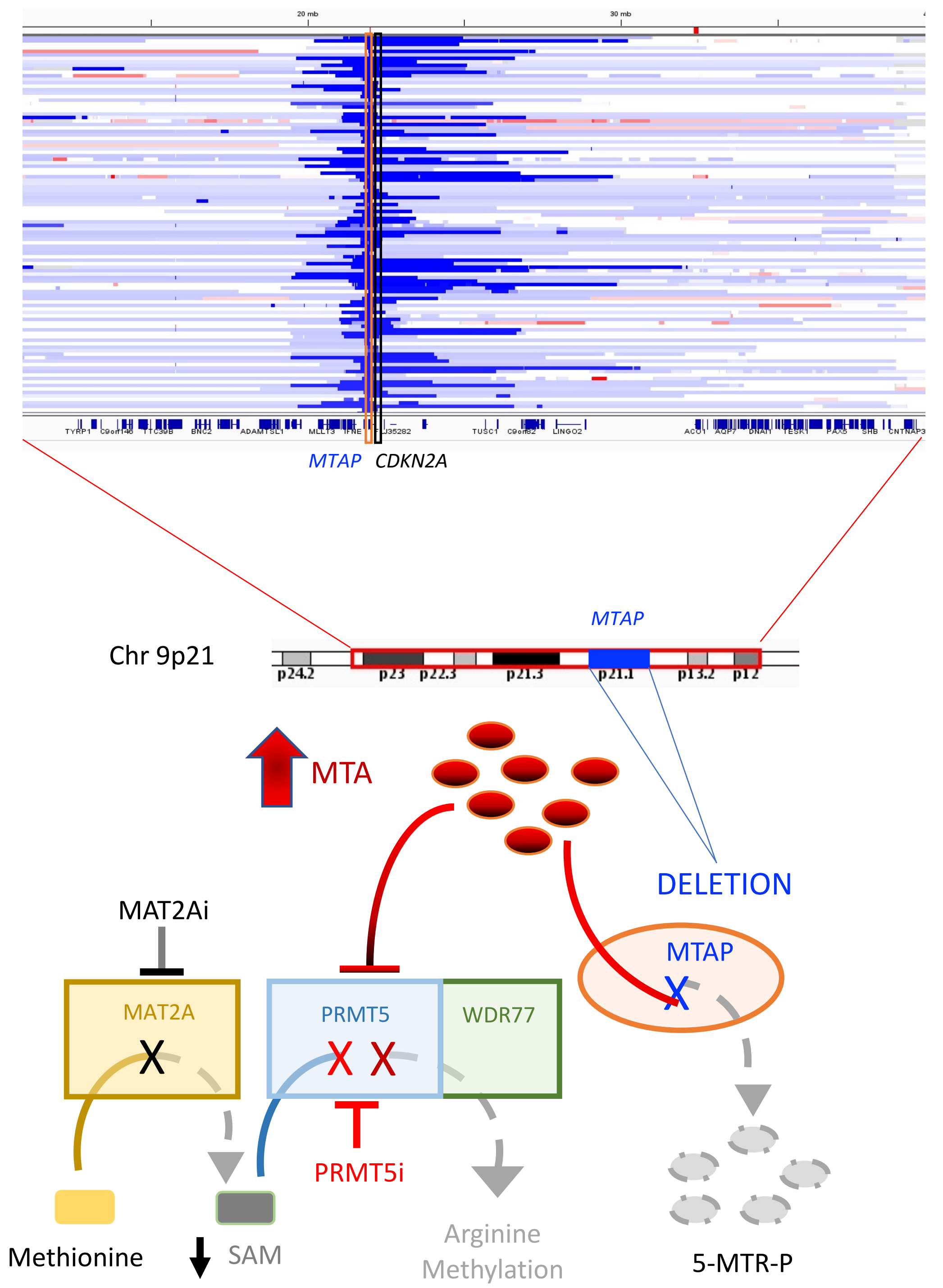

Supplementary Figure 1: Genomic deletion of *MTAP* as part of the 9p21 locus and the associated metabolic vulnerabilities. Genomic copy-number data (dark blue: bi-allelic or homozygous deletion) from a broad panel of tumors, from The Cancer Genome Atlas database, around the tumor suppressor *CDKN2A* in the 9p21 locus. Each strip in the y-axis represents a single tumor, with dark blue showing homozygous deletion regions at the specific chromosomal location (x-axis). The co-deletion of *MTAP* is expected to result in MTA accumulation. MTA is a structural analog of SAM, and its accumulation can sensitize cells with *MTAP*-deletions to either MAT2A inhibitors[1] or inhibition of PRMT5[2,3] and even PRMT1[4,5] by acting as a SAM-competitive inhibitor. This competitive inhibition is likely a physiologic regulatory feedback mechanism to prevent the overproduction of polyamines.

Supplementary Figure 2

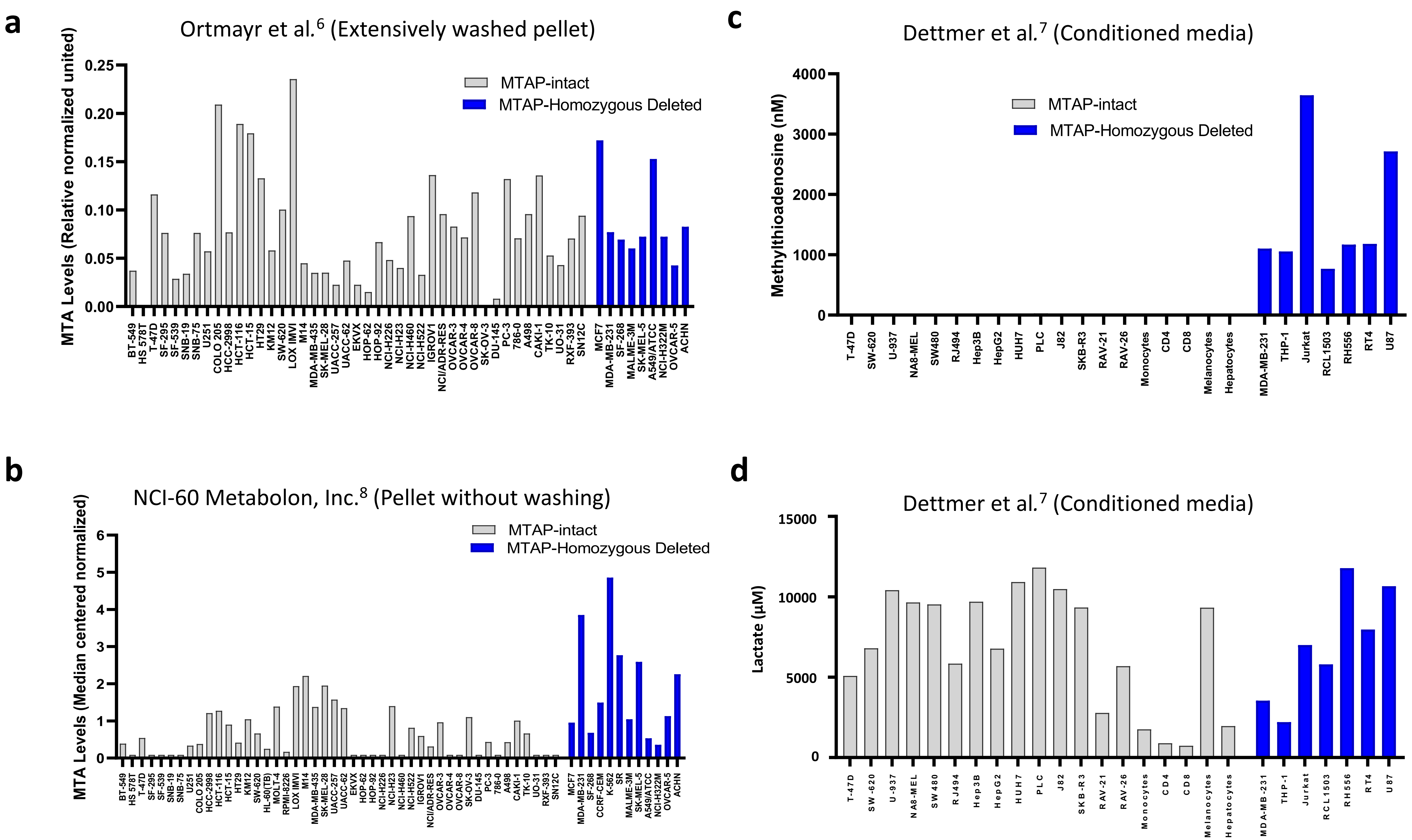

Supplementary Figure 2: Measurements of MTA levels of *MTAP*-intact versus deleted cancer cell lines vary depending on how well intracellular versus secreted metabolites are separated during sample preparation. Methylthioadenosine (MTA) levels from three different studies (Ortmayr et al., 2019[6]; Dettmer et al., 2013[7]; Su et al., 2011[8]) are compared. MTA levels from the supplementary data of those studies are replotted, with each bar representing a single cell line's levels. Most of these cell lines are part of the NCI-60 panel (cell line name in the x-axis, *MTAP*-deleted cell lines in blue). (**a**) Ortmeyer et al.[6] study specified that the cell pellets were washed extensively, while (**b**) NCI-60 data performed by Metabolon, Inc. did not. Even though both studies were conducted with the same set of cell lines and under the same culturing conditions, the NCI-60 Metabolon, Inc. data show that the *MTAP*-deleted cell lines have a ~3-fold higher MTA levels (0.62± 0.09 vs. 1.87 ±0.4, P=0.01, t-test). In contrast, the Ortmayr data show no difference (0.077 ± 0.008 vs. 0.089 ± 0.014, n.s.). (**c**) The study by Dettmer et al.[7] used conditioned media, reflecting MTA levels that are, i.e., exclusively extracellular. The levels of MTA are >100-fold higher in *MTAP*-deleted vs *MTAP*-intact cell lines (7.9 ± 1.7 (n=19) vs 1663 ± 408 (n=7), P<0.007 t-test). (**d**) As a control, the lactate levels in the conditioned media from the Dettmer et al. study are shown. No difference is seen between the *MTAP* deleted and intact groups. The MTA levels are given as average; the t-test is unpaired, two tails, unequal variance.

Supplementary Figure 3

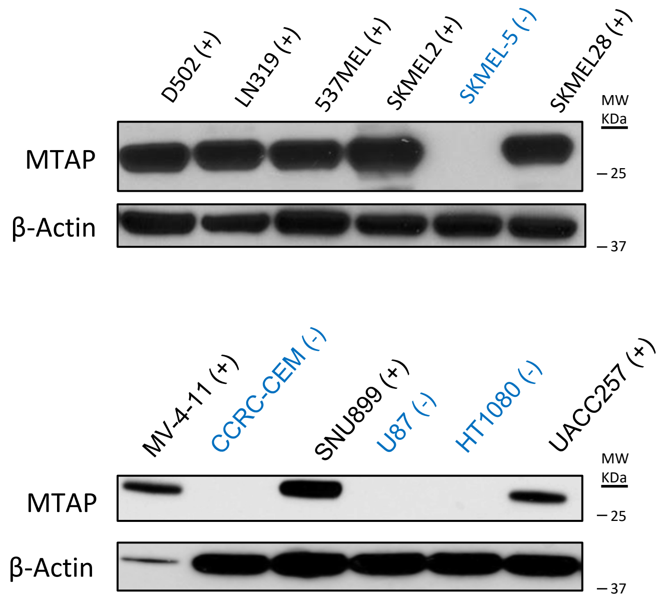

Supplementary Figure 3: ***MTAP*** **status of the panel of cell lines used in this study is confirmed by immunoblotting, repeated independently once.**

Supplementary Figure 4

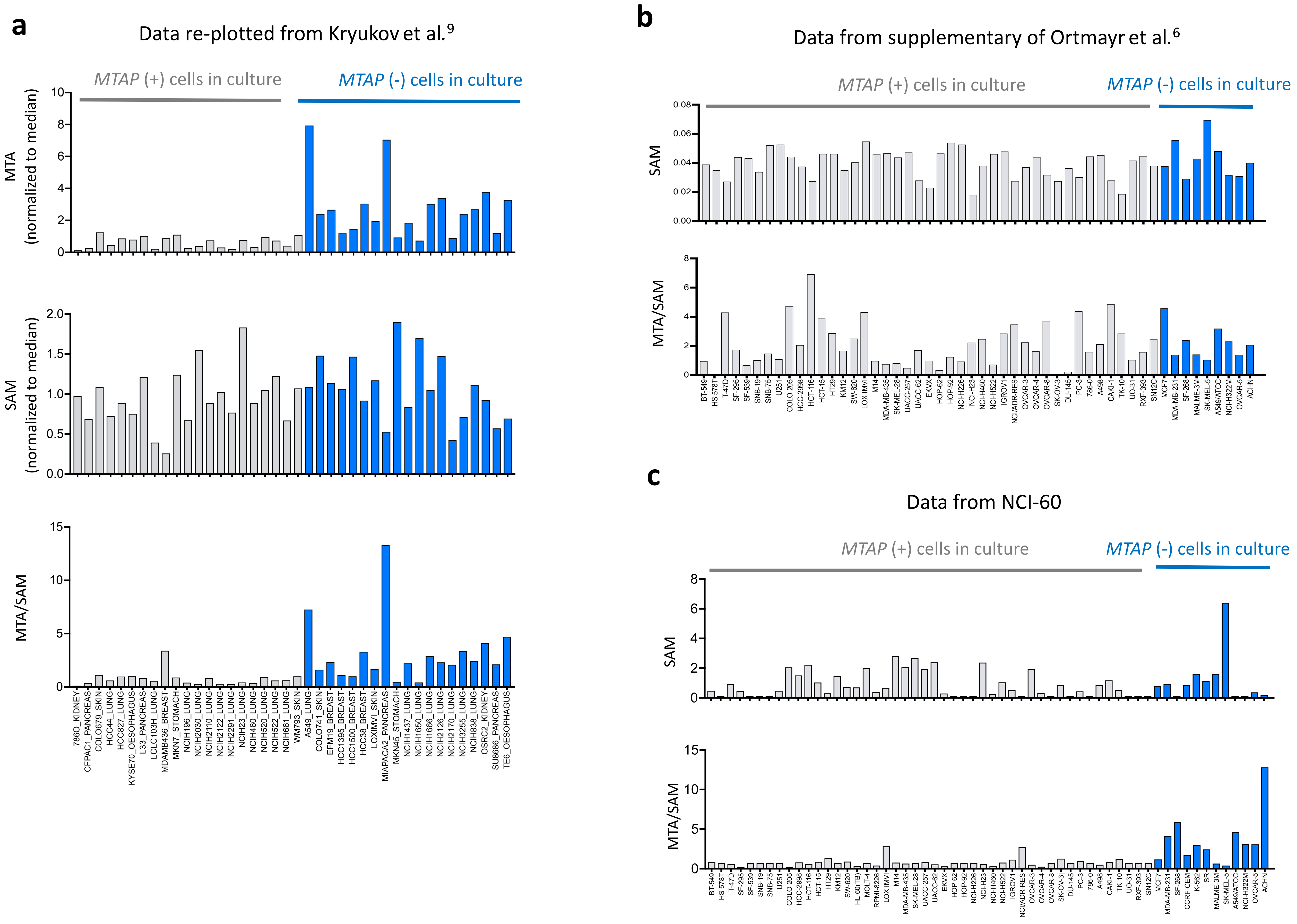

Supplementary Figure 4: : Intracellular SAM as a normalization factor for MTA across different cell culture experiments. Metabolites levels of cells in culture for three different studies indicating that the levels of SAM are not different between *MTAP*-deleted (*MTAP* -) and intact cells (*MTAP* +). (**a**) The levels of MTA (mean *MTAP*-deleted 0.63, mean *MTAP*-intact 2.73, p-value 0.00015), SAM (mean *MTAP*-deleted 0.94, mean *MTAP*-intact 1.06, p-value 0.33) and the ratio of MTA/SAM (mean *MTAP*-deleted 0.76, mean *MTAP*-intact 3.09, p-value 0.003) for cells in culture replotted from the supplementary data of Kryukov et al., 2016 [9]. (**b**) Data from the supplementary of Ortmayr et al.[6] specified that cell pellets were washed extensively. The MTA levels for this data set are shown in **supplementary figure 2a** with no significant difference between *MTAP* deleted and intact groups (1.15-fold higher MTA levels in *MTAP* deleted cells compared to wild-type, p=0.49). SAM and MTA/SAM levels are 1.1-fold (p = 0.47 for SAM and p = 0.77 for MTA/SAM, t-test) higher in *MTAP*-deleted cells versus wildtype. (**C**) Metabolomic data from NCI-60 where the cell pellets were not washed extensively. The levels of SAM and MTA/SAM are 1.4-fold (p = 0.53, t-test) and 4.5-fold (p = 0.01, t-test) higher in *MTAP*-deleted cells versus wildtype. MTA levels are shown in the **supplementary figure 2b,** where MTA levels 3-fold higher (p = 0.01) in *MTAP*-deleted cells versus wild-type. The unpaired two-tailed student t-test with unequal variance was used to calculate p-values.
This figure illustrates that MTA's ratio to SAM has a similar trend as MTA levels; thus, it is a suitable metabolite for normalization purposes.

Supplementary Figure 5

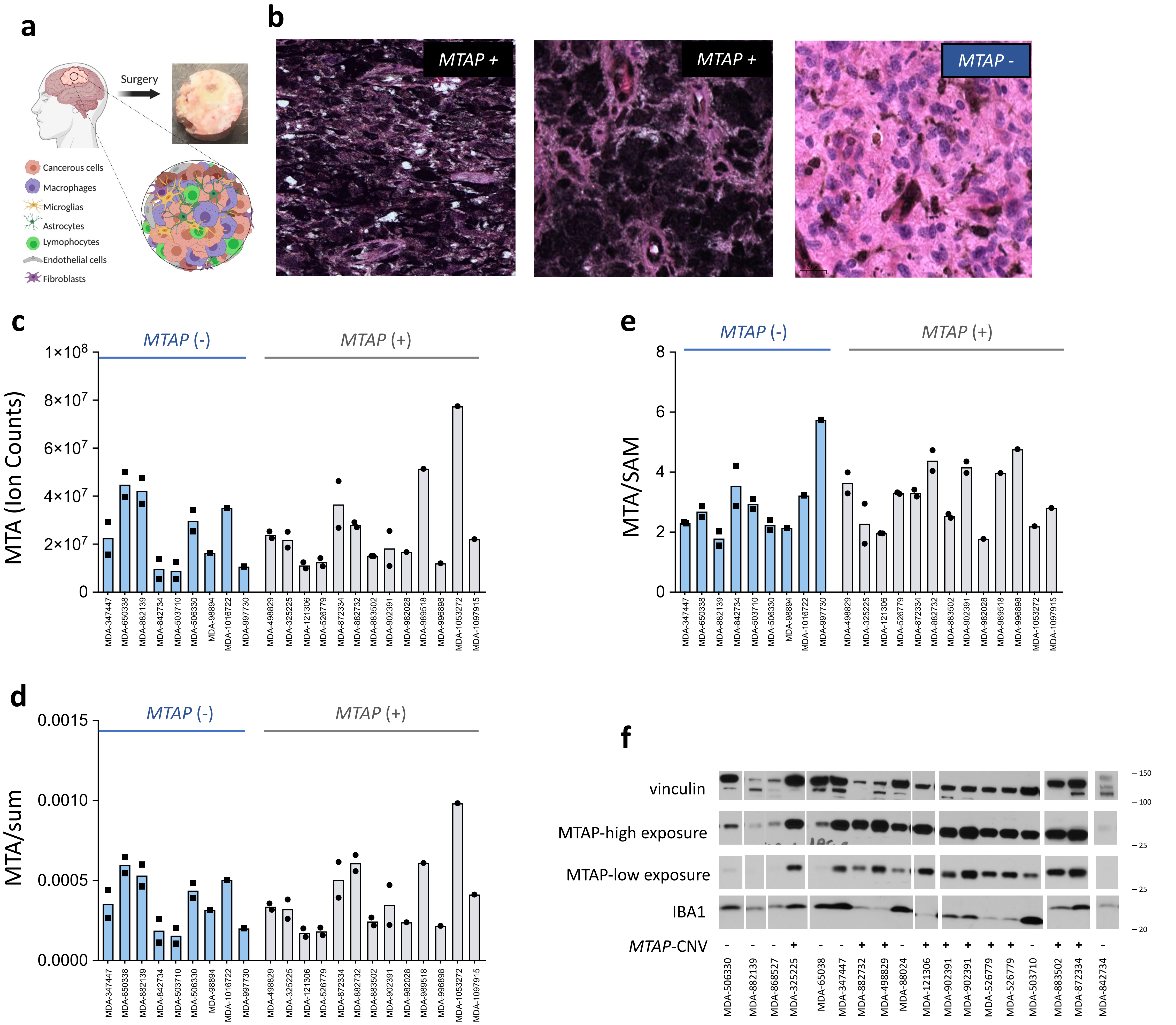

Supplementary Figure 5: No significant elevation of MTA in *MTAP*-deleted primary resected GBM tumors in an independent data set. (**a**) Flash-frozen primary resected GBM tumors (heterogeneous mix of transformed glioma cells and non-malignant stromal cells) were prepared for IHC, mass spectroscopy, and Western blotting. (**b**) *MTAP* deletion status of tumors was confirmed by immunohistochemistry using a validated MTAP antibody. Representative cases of *MTAP* positive and *MTAP* negative GBM tumors are shown. Non-malignant *MTAP*-intact cells are stained positive in the *MTAP*-deleted case. (**c**) MTA levels (mean, N = 2 or 1 biological replicates) in *MTAP*-deleted (blue) versus *MTAP*-intact (gray) GBM tumors, using the Metabolon, Inc. platform. There is a 1.14-fold increase in the median MTA levels in *MTAP*-deleted tumors compared to intact tumors (p = 0.9, unpaired 2-tailed t-test with unequal variance). (**d**, **e**) Same data (mean, N = 2 or 1 biological replicates) but expressed as a ratio of each tumor's total ion count for sample loading normalization (**d**) and as a ratio to SAM levels to account for upregulation in the methionine salvage pathway for each tumor (**e**). The median MTA/sum and MTA/SAM fold change in *MTAP*-deleted tumors compared to intact tumors are 1.23 (p=0.9) and 0.8 (p=0.3) respectively. P-values calculated using unpaired 2-tailed t-test with unequal variance. (**f**) The levels of vinculin, MTAP, and IBA1 (myeloid marker, microglia/macrophages) in some of the tumors, repeated once. Non-malignant MTAP-expressing cells (e.g., IBA1-expressing cells) drive the no-zero MTAP expression in the Western blot of homozygous *MTAP*-deleted GBM tumors.

Supplementary Figure 6

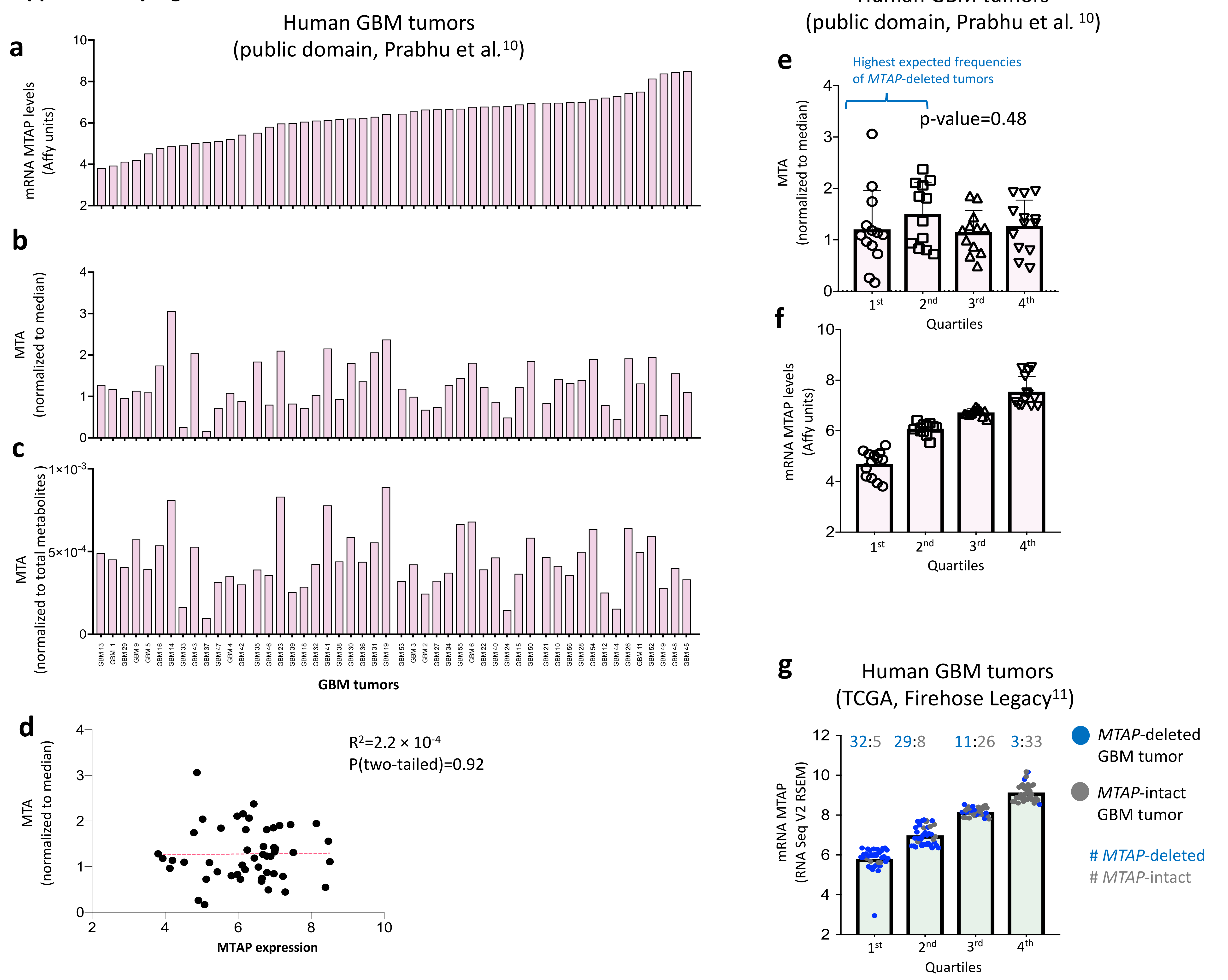

Supplementary Figure 6: No correlation between MTAP expression and MTA levels in primary human GBM tumors in an independent dataset. (a-f) Data replotted from Prabhu et al., Neuro. Oncol, 2019[10], where metabolomic profiling was performed by Metabolon Inc. and gene expression analysis was performed by Affymetrix Human expression array. (**a**) MTAP mRNA levels of 50 primary human GBM tumors sorted from low to high, (**b**) MTA levels normalized to the median, and (**c**) MTA levels as a ratio of total ion count to account for differential loading, sorted based on their MTAP expression in **a**. (**d**) The scatter plot of MTA levels versus MTAP expression with an insignificant coefficient of correlation highlighting no matter how we analyze these data, the results came insignificant. (**e, f**) MTA and MTAP mRNA levels (mean + SD, N = 12 or 13 biologically independent tumors) among the same 50 human primary GBM tumors sorted based on MTAP expression and divided into four quartiles. The frequency of *MTAP* deletion in GBM tumors is about 50%. That said, in the population of 50 tumors, it is much more likely that tumors with low bulk MTAP expression belong to the 50% of the group with homozygous *MTAP*-deletion vs. 50% *MTAP*-intact. In other words, the vast majority of homozygous *MTAP*-deleted tumors fall in the first and second quartiles of low MTAP expression. In contrast, the bulk of *MTAP*-intact tumors fall into the third and fourth quartiles of higher mRNA MTAP expression. Comparing MTA levels between quartiles suggests no significant elevation of MTA in quartiles with low MTAP expression vs. high expression (mean MTA levels are 1.20, 1.50, 1.15, and 1.27 for each quartile, $p = 0.48$, Anova: single factor). This figure supports our conclusion from tumors with known *MTAP*-deletion status (**Figure 2** and **Supplementary Figure 5**). (**g**) MTAP mRNA levels (mean + SD, N = 37 or 36 biologically independent samples) of 147 GBM tumors with known *MTAP*-deletion status from the TCGA dataset through cBioPortal[12,13] (Firehose Legacy, with source of data are available at http://gdac.broadinstitute.org/runs/stddata__2016_01_28/data/GBM/20160128/) indicating that the majority (not all) of homozygous *MTAP*-deleted tumors fall in the first and seconds quartiles of low MTAP expression. MTAP mRNA expression was sorted from low to high and divided into four quartiles. Since the frequency of *MTAP* deletion in GBM is about 50%, most tumors in the two lowest quartiles of MTAP mRNA expression are homozygous *MTAP*-deleted. In contrast, the majority of the tumors in the last two quartiles are *MTAP*-intact.

Supplementary Figure 7

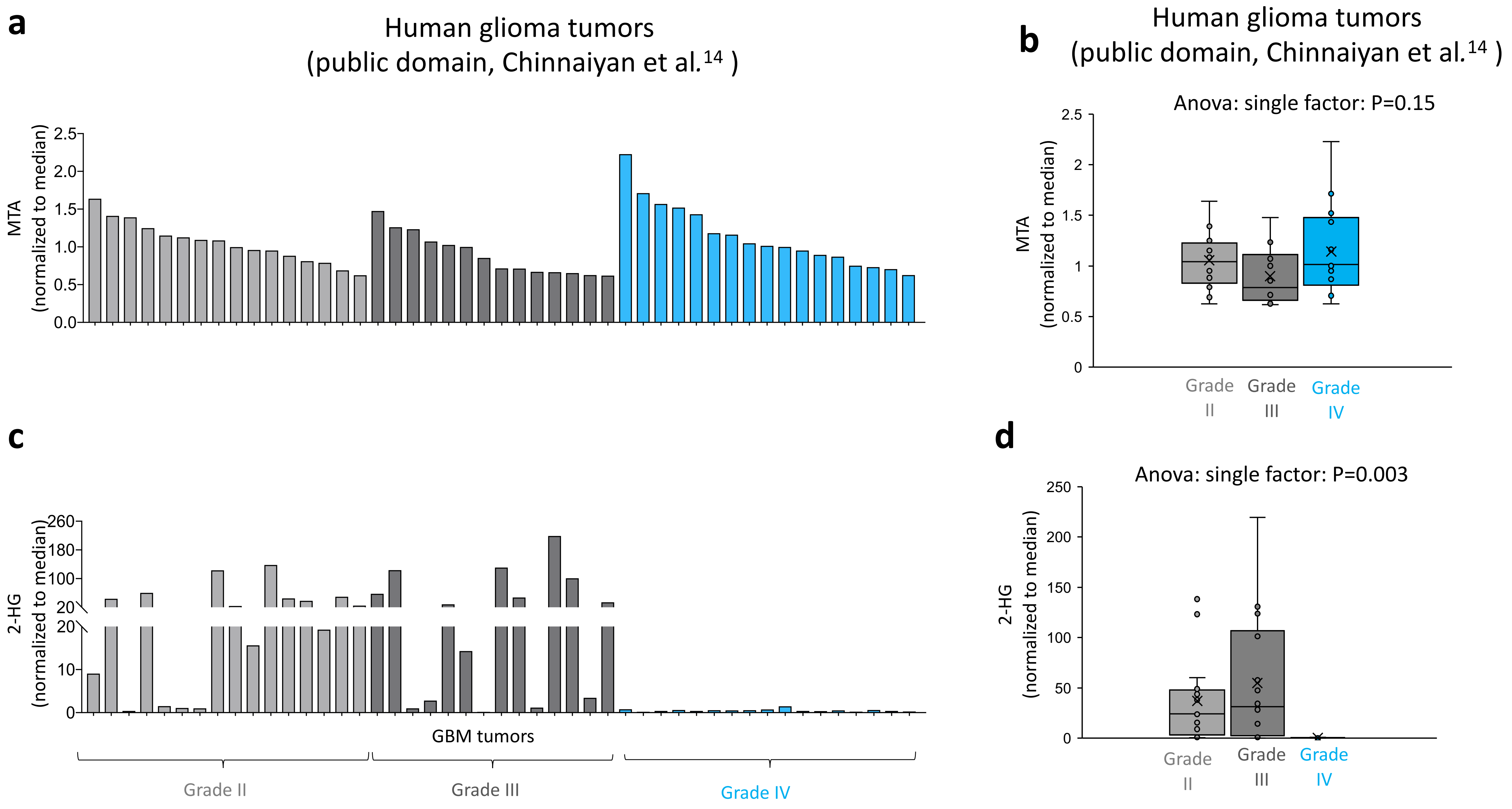

Supplementary Figure 7: Minimal and insignificant elevation of MTA in primary grade IV (GBM) compared to low-grade gliomas. MTA and 2-HG levels in a series of primary grade II and III gliomas (gray) and grade IV glioma (GBM, blue) from a global metabolome profile using the Metabolon, Inc. platform, plotted from Supplementary data from Chinnaiyan et al. Cancer Res. 2012. [14] (**a**) The bar graph of MTA levels for different grades of glioma tumors where each bar represents MTA's level for each tumor. (**b**) The box and whisker graph of data plotted in **a** comparing MTA levels in grade II (N = 16 biologically independent samples), III (N = 13 biologically independent samples), and IV (N = 17 biologically independent samples) gliomas. While the *MTAP*-deletion status of individual tumors in that study is not given, ~50% of GBM tumors have *MTAP*-homozygous deletions. In comparison, less than 3% of grade II glioma are *MTAP* deleted. Thus, given the stark difference in the frequency of *MTAP* deletion in low-grade glioma and GBM, we expect a higher level of MTA in GBM compared to other groups. However, we observed minimal and insignificant elevation of MTA in primary GBM tumors compared to others (mean MTA levels of 1.05, 0.90, and 1.14 for grade II, III, and IV gliomas, respectively, $p = 0.15$, Anova: single factor). (**c**) The bar graph and (**d**) the box and whisker graph of 2-HG levels in the same dataset with mean 2-HG levels of 37.1, 54.7, and 0.5 for grade II, III, and IV gliomas respectively, $p = 0.003$, Anova: single factor. The significant elevation of 2-HG in low-grade gliomas (with *IDH* frequency mutation of ~80%) compared to the primary GBM tumors (with *IDH* frequency mutation of ~10%) highlights what a massive increase in a specific metabolite by a specific genetic event looks like, and how it contrasts with the marginal increases in MTA in *MTAP*-deleted tumors. Box plots in **b** and **d** are defined as: the middle line is the median, and the lower and upper bounds of box represents the first quartile (Q1) and third quartile (Q3). The upper and lower whiskers (maximum and minimum) defined as Q3+1.5*IQR and Q1-1.5*IQR where IQR is the inter-quartile range. Data beyond the limits of whisker are considered as outliers.

Supplementary Figure 8

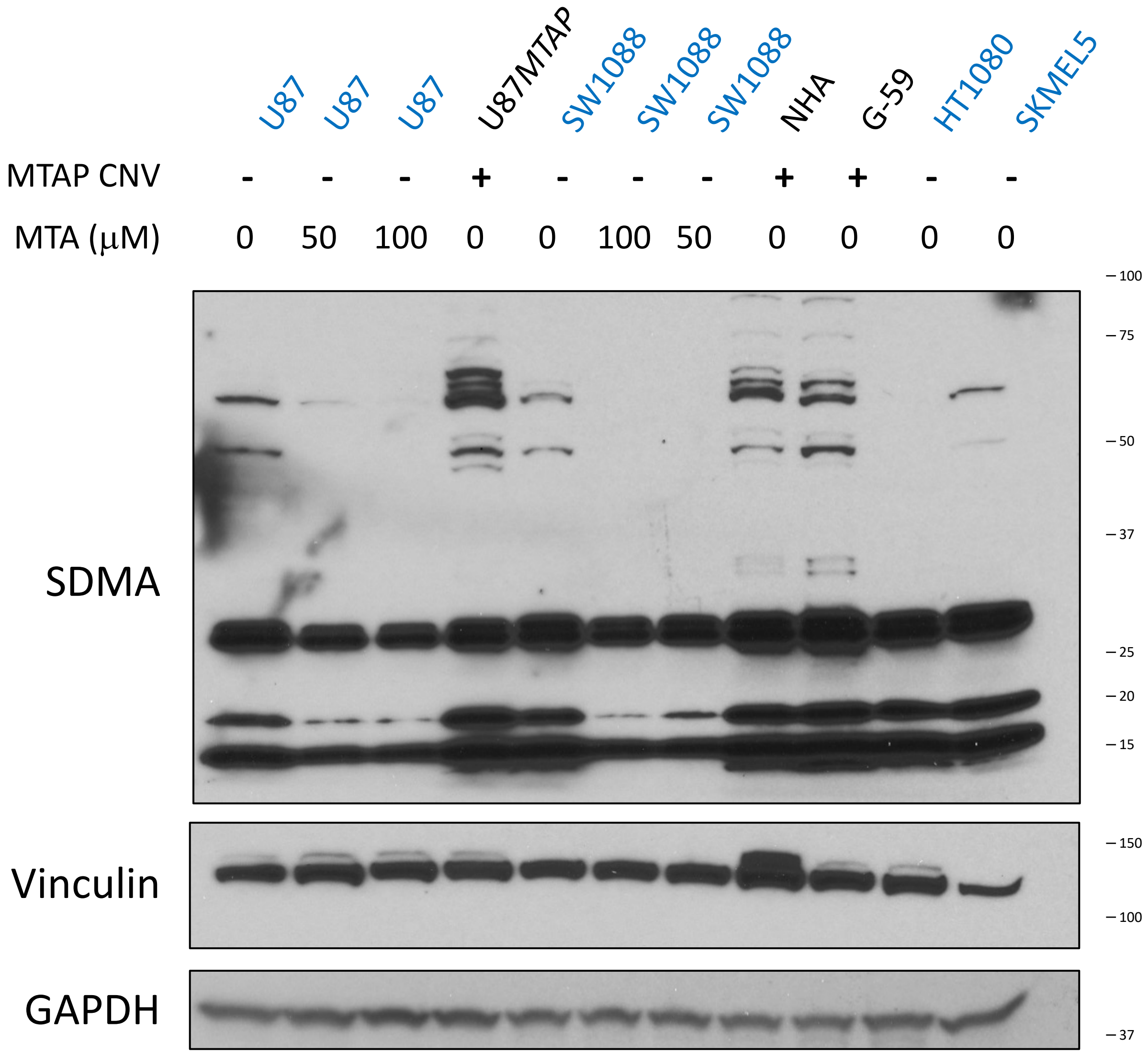

Supplementary Figure 8: Lower levels of SDMA in *MTAP*-deleted cells compared to the *MTAP*-intact cells in culture. PRMT5 mediates the formation of symmetric dimethylarginine (SDMA); thus, its inhibition results in less SDMA levels. Compared to the *MTAP* wild-type cells, SDMA levels are less in *MTAP* deleted cells. Also, the treatment of cells with MTA (50 μM and 100 μM) for two days result in more reduction in SDMA levels.

Supplementary Figure 9

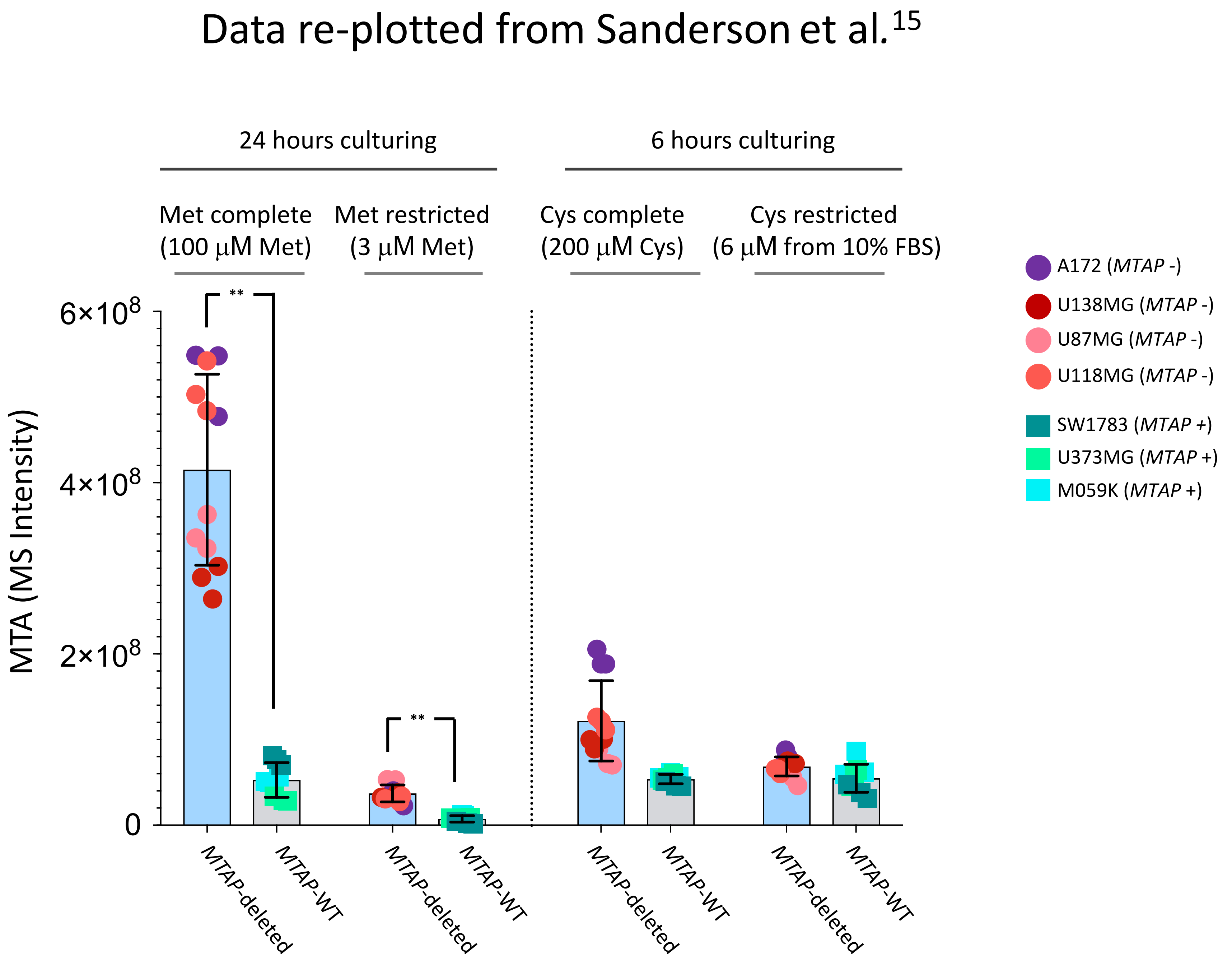

Supplementary Figure 9: Disparate effects of methionine and cysteine deficiency on MTA levels in MTAP deleted versus intact cancer cells. Data re-plotted from Sanderson et al., Science Advances, 2019[15]. The comparison of MTA levels in the *MTAP*-deleted (mean +/- SD, N = 3 biological replicates and 4 independent cell lines) and WT glioma cells (mean +/- SD, N = 3 biological replicates and 3 independent cell lines) when cells cultured in the standard formulation of RPMI (100 μM methionine and 200 μM cysteine) versus restricted methionine (3 μM) or restricted cysteine (6 μM) conditions. MTA levels decreased in *MTAP*-deleted and WT cells when cells were cultured in extremely low methionine conditions. However, the difference in MTA levels between the *MTAP*-deleted cells and WT remains significant – more MTA in *MTAP*-deleted cells than WT. This data suggest that MTA accumulated inside *MTAP*-deleted cells even when cells were cultured in the restricted methionine condition. While methionine deficiency can lower MTA levels in cells but could not explain why we do not observe the significant difference between *MTAP*-deleted and WT human GBM tumors. **P=0.02, significant values are obtained using multiple t-test analysis with Benferroni correction.

On the other hand, culturing cells in the restricted cysteine media decreased MTA levels only in the *MTAP*-deleted glioma cells, but not *MTAP*-WT cells. This may indicate that cysteine deprivation inside tumors could potentially cause no MTA accumulation in *MTAP*-deleted human GBM tumors. However, we note two points, (i) the cysteine and cystine's average concentrations in human plasma (34 μM and 48 μM) are much higher than their concentration in the cysteine restricted condition (0 μM and 6 μM); and (ii) Sanderson *et al.*[1] used a shorter culture time (6 hours) for the restricted cysteine experiments due to the toxicity caused by the cysteine deprivation. Since the accumulation of MTA is time-dependent (more MTA accumulates with increasing culture time, **Figure 1F**), it could be possible that the difference in MTA levels between *MTAP*-deleted and WT cells cultured in restricted cysteine conditions became more significant if cells were grown for a longer time (i.e., 24 hours).

Supplementary Figure 10

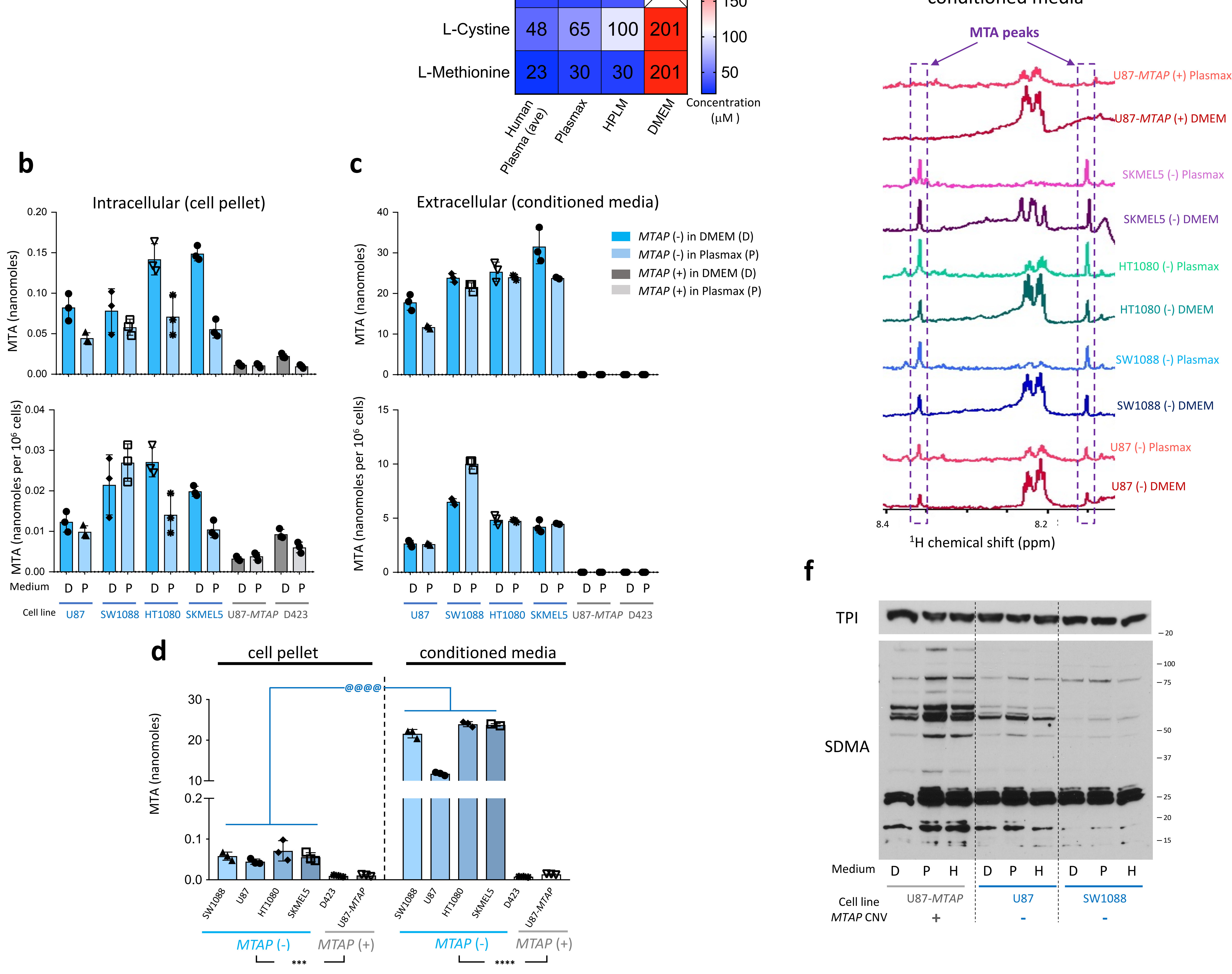

Supplementary Figure 10: Comparison between intracellular and extracellular MTA levels in cells cultured in DMEM versus Plasmax. (a) Comparison between the concentrations of methionine, cysteine, and cystine in historic medium (DMEM) and physiological media (Plasmax and HPLM) and human plasma (concentrations expressed in micromolar). The formulations of physiological media such as Plasmax better recapitulate the human plasma and tumors' nutrition environment. Historic media contain a much higher concentration of some metabolites, i.e., methionine and cystine, compare to human plasma, but they lack many other metabolites, i.e., cysteine. *MTAP*-deleted and WT cells were cultured in DMEM, and Plasmax supplemented with 2.5% FBS. After three days, cells and media were harvested for mass-spectroscopy, NMR, and western blotting. DMEM, Plasmax, and HPLM are indicated as D, P, and H, respectively. (**b and c)** Intracellular and extracellular levels of MTA (N=3 biological replicates, mean +/- SD) of cells cultured in DMEM vs. Plasmax using mass spectroscopy. The average MTA levels in the cell pellet or conditioned media of the *MTAP*-deleted cells do not differ significantly between DMEM and Plasmax. (**d**) Absolute quantification of MTA (N=3 biological replicates, mean +/- SD) in cell pellet and conditioned media of cells cultured in Plasmax. The amount of MTA recovered from conditioned media of *MTAP*-deleted cells cultured in Plasmax is significantly greater than that recovered from the cell pellets, recapitulate the cell culture data in DMEM. Thus, *MTAP*-deleted cells cultured in Plasmax secrete MTA to the conditioned media resulting in the predominant extracellular presence of MTA. Significant values are indicated as @@@@P=8×10^{-11}, ***P = 10^{-5}, and ****P = 8×10^{-7} using multiple t-test with Bonferroni correction. (**e**) Further validation of accumulation extracellular MTA in DMEM vs. Plasmax by NMR. (**f**) SDMA levels of *MTAP*-deleted and WT glioma cells when cultured in DMEM vs. Plasamax and HPLM, repeated once. No significant difference is observed in the SDMA levels of cells cultured in traditional medium and physiological ones.

Supplementary Figure 11

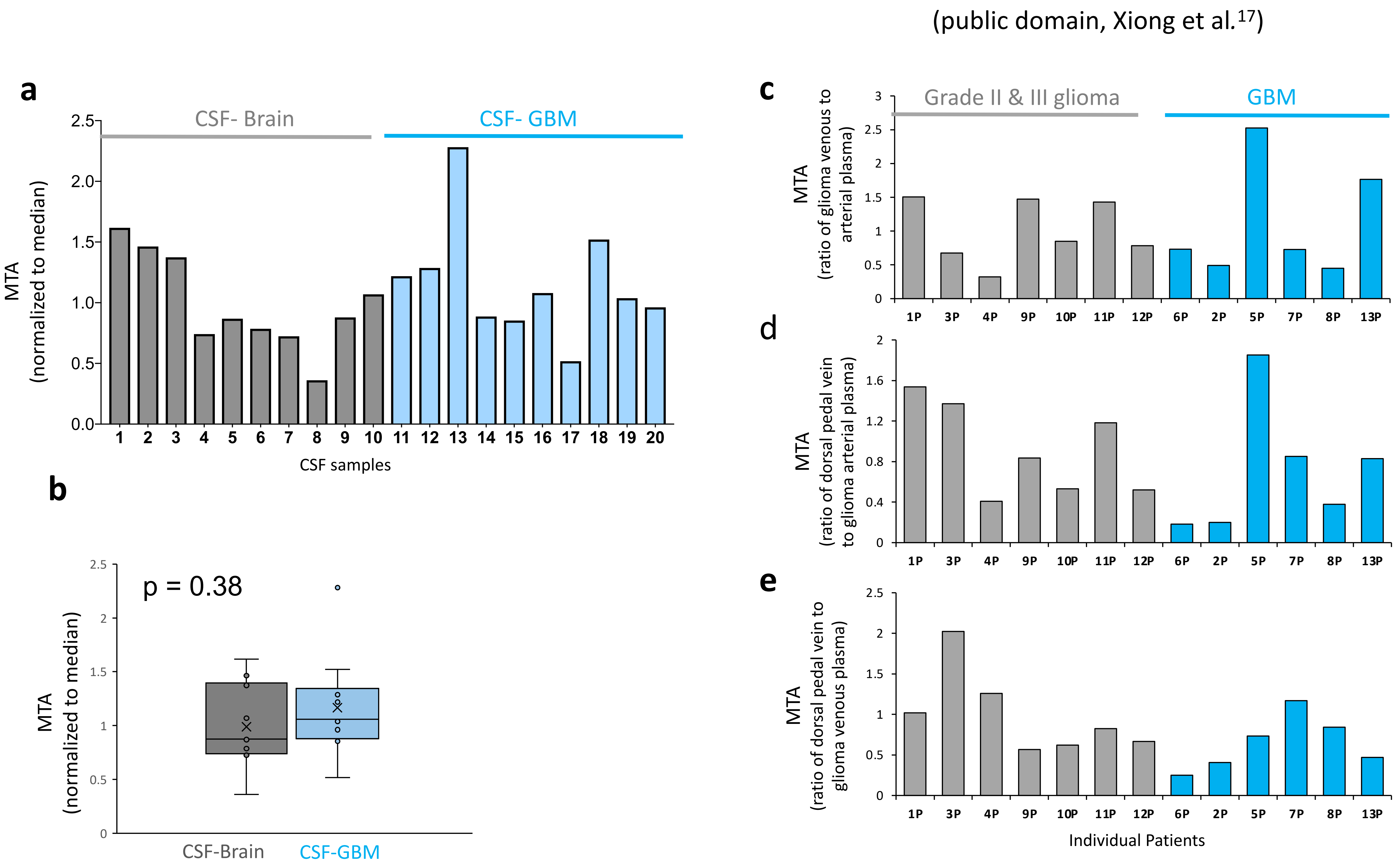

Supplementary Figure 11: No significant elevation of MTA in cerebrospinal fluid (CSF) or venous plasma of patients with GBM. The X-axis represents samples obtained from different patients. (**a, b**) Replotted data (file name: 011210QssCSF2HGnormGBM.txt) is kindly shared by John Asara using the BIDMC metabolomics platform, originally published in Locasale et al[16]. (**a**) MTA levels in CSF were collected from normal brain and GBM, where each bar represents the level of MTA normalized to the median for each individual sample. (**b**) MTA levels in CSF collected from the brain (CSF-Brain) versus GBM (CSF-GBM). Data are represented as boxplots where the middle line is the median, and the lower and upper bounds of box represents the first quartile (Q1) and third quartile (Q3). The upper and lower whiskers (maximum and minimum) defined as Q3+1.5*IQR and Q1-1.5*IQR where IQR is the inter-quartile range. Data beyond the limits of whisker are considered as outliers. Given the extracellular accumulation of MTA in vitro and 50% frequency of *MTAP* deletion in GBM, we expect to see higher MTA levels in the CSF-GBM group than CSF-Brain. However, no statistically significant difference was observed between each group's means (the mean MTA level is 1.17 higher in CSF collected from GBM than CSF collected from normal brain, $p = 0.38$, unpaired 2-tailed t-test with unequal variance). These data may suggest that secreted MTA from *MTAP*-deleted cells is further metabolized by *MTAP*-wildtype cells inside the tumor. (**c**-**e**) Data obtained from the supplementary of Xiong et al., Nat. Commun, 2020[17]. (**c**) To see if *MTAP* deleted tumors secrete more MTA into the bloodstream, we looked at the MTA levels as a ratio of venous to arterial plasma obtained from downstream and upstream of the glioma. High levels of the venous to arterial plasma ratio for MTA indicate that a GBM produced/secreted more MTA into the downstream vein. Given the frequency of *MTAP*-deletion in GBM, we expect to have one or two GBM tumors be *MTAP* deleted. One patient (5P) has a high venous to arterial plasma ratio for MTA in this dataset. However, (**d**) the ratio of a dorsal pedal vein to glioma arterial plasma for MTA is high for the same patient (5P), which indicates that high levels of MTA are also produced by cells in the patient's foot (*MTAP*-wildtype). (**e**) The MTA levels as the ratio of a dorsal pedal vein to glioma venous plasma (MTA levels produced by cells in foot versus cells in a tumor), where no outlier was observed for GBM tumors. These data, **c**-**e**, and MTA levels in CSF and primary human GBM tumors suggest that secreted MTA is further metabolized by *MTAP*-wildtype cells inside tumors.

Supplementary Figure 12

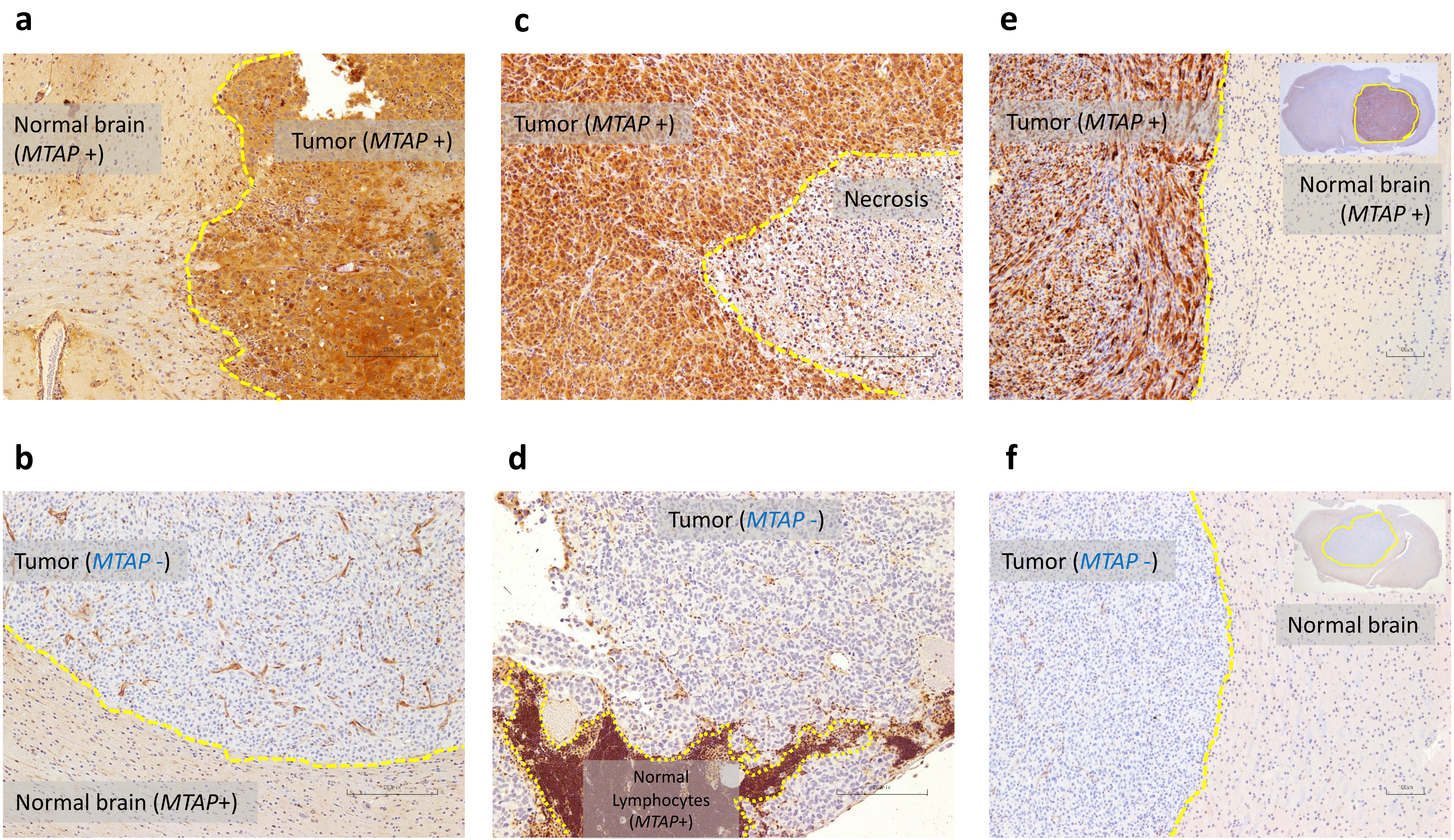

Supplementary Figure 12: Validation of an MTAP rabbit monoclonal antibody to detect *MTAP*-deleted tumors by IHC on FFPE sections. Xenografted tumors (S.C.: a, b; intracranial: c, d, e, f) differencing in *MTAP*-deletion status (**a**, D423, MTAP intact, **b,** U87, *MTAP*-homozygous deleted, **c**, NB1, *MTAP*-intact, **d**, SKMEL5, *MTAP*-homozygous deleted, **e**, U87 pCMV MTAP; *MTAP*-rescued, **f,** U87 *MTAP*-homozygous deleted) were grown in immunocompromised mice and FFPE sections generated. IHC was performed with rabbit monoclonal anti-MTAP (ab126623; EPR6892) and slides developed by NOVA red (red-brown staining indicating MTAP presence) and counterstained by hematoxylin (blue, nuclei). Tumor boundaries are shown in yellow. Note the clear correspondence between *MTAP* genomic status and staining intensity in tumors, with the complete absence of staining in *MTAP*-deleted tumors. The MTAP antibody yields intense staining pretty much in all cells except those with *MTAP*-deletions and regions of necrosis. This fully validates that this genuinely detects MTAP protein by IHC in FFPE sections. At least 12 xenografted tumors were stained with MTAP using this method.

Supplementary Figure 13

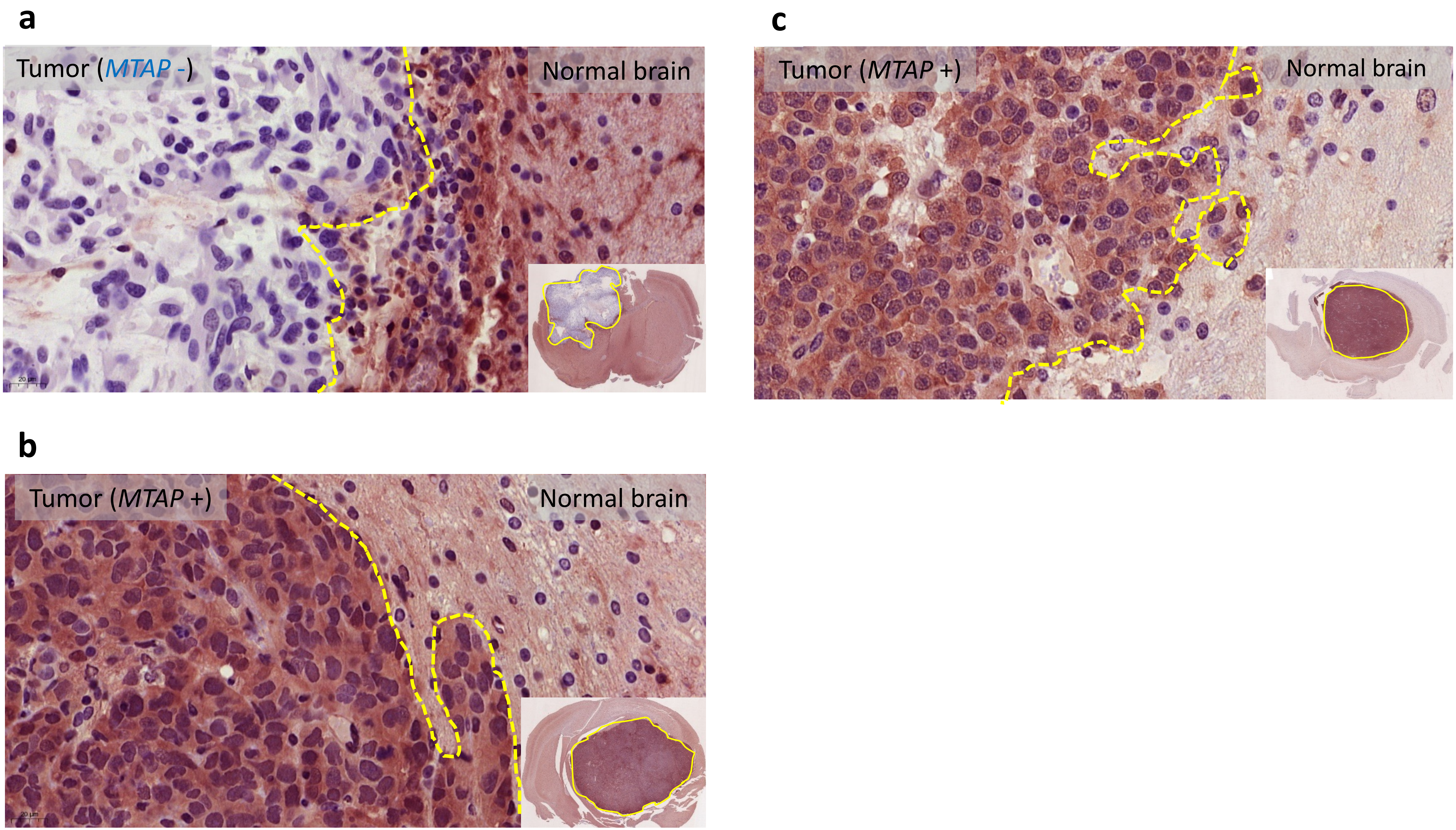

Supplementary Figure 13: IHC Staining with the MTAP monoclonal antibody remains specific even at longer exposures. FFPE sections of xenografted tumors generated from glioma cell lines differing in *MTAP* deletion status were stained with anti-MTAP rabbit monoclonal (ab126623) and developed with NOVAred (brown/red) and counterstained with hematoxylin (nuclei, blue). (**a**) Gli56 (*MTAP* -; deleted); (**b**) NB1, (**c**) D423 (*MTAP* +; intact); The exposure of the developer was increased compared to experiments in Fig S12, to determine whether non-specific background staining would start to occur in *MTAP*-deleted tumors; no evidence of this is present (**a**), fully validating the use of this antibody to evaluate *MTAP*-deletion status in human FFPE GBM sections. At least 6 xenografted tumors were stained with MTAP using this method.

Supplementary Figure 14

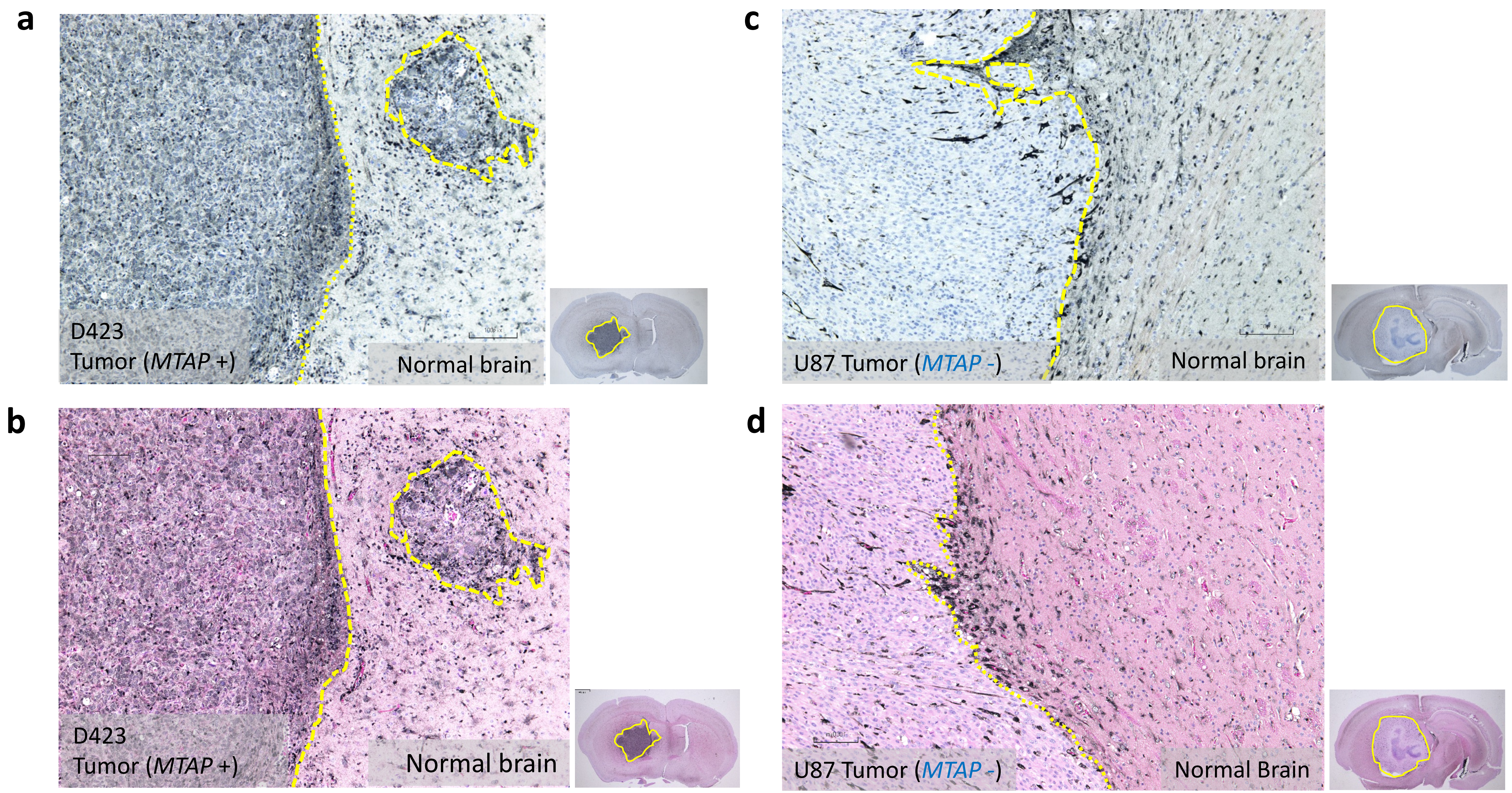

Supplementary Figure 14: MTAP IHC developed by EnzMet with or without Eosin counterstain yields increased histological resolution and shows minimal *MTAP*-WT stromal infiltration in xenografted *MTAP*-deleted tumors. (**a, b**) Intracranial xenografts generated with D423 (*MTAP*-WT) glioma cells, **c, d**: with the U87 (*MTAP*-deleted) glioma cell lines. IHC staining with anti-MTAP (ab126623**)** was performed as in Fig S2 and Fig S3, except that instead of NovaRed, antibodies were developed using the EnzMet silver developer (black). Areas of immunopositivity (MTAP presence) are black rather than Red/brown in NovaRED. Unlike NovaRed or DAB, EnzMet staining is not washed out by ethanol, allowing Eosin staining for a higher level of histological detail to be discernable. (**a, c**) stained with anti-MTAP and developed by EnzyMet with Hematoxylin counterstain and **b, d** with additional Eosin counterstain. The advantage of EnzMet is a lower background and higher ultrastructural resolution and tolerance to Eosin counterstain. We have used this developer for the FFPE primary human GBM studies. U87 glioma cells stain negative for MTAP, with normal brain parenchyma staining strongly; a small amount of positive MTAP staining is evident in tumors, consisting of non-malignant stromal cells: endothelial, fibroblasts, microglia. At least 8 xenografted tumors were stained with MTAP using this method.

Supplementary Figure 15

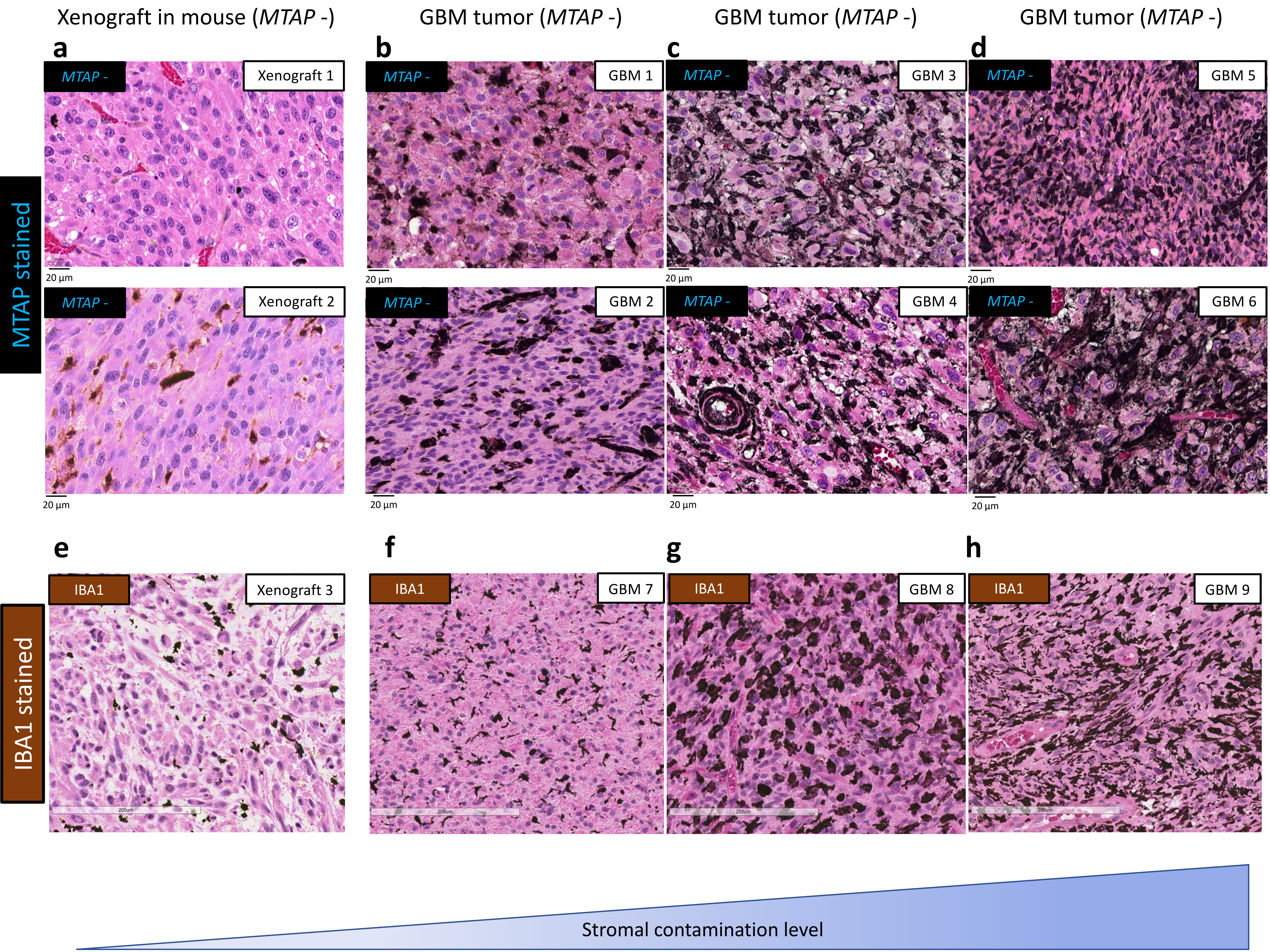

Supplementary Figure 15: Negligible stromal infiltration in human xenografts in mice compared to primary GBM tumors. FFPE sections were stained against MTAP (**a-d**) and IBA1 (**e-h,** microglia marker) antibodies (black) and counterstained with hematoxylin (nuclei, blue) eosin (protein, pink). (**a**) *MTAP*-deleted intracranial xenografted in mice (U87), (**b-d**) *MTAP*-deleted human GBM tumors with varying degrees of stromal content (minimal to extreme stromal content). The degree of *MTAP*-positive stromal infiltration was much more significant in the primary human GBM tumors than xenografts. (**e**) *MTAP*-deleted intracranial xenografted in mice (Gli56), (**f-h**) *MTAP*-deleted human GBM tumors with varying degrees of IBA1-positive cells (stromal content). The amount of IBA1-positive (myeloid) cells are dramatically higher in human GBM tumors (even in an example of “low” stromal content) than xenografts. At least 50 human tumors were stained with MTAP and IBA1 antibodies.

Supplementary Figure 16

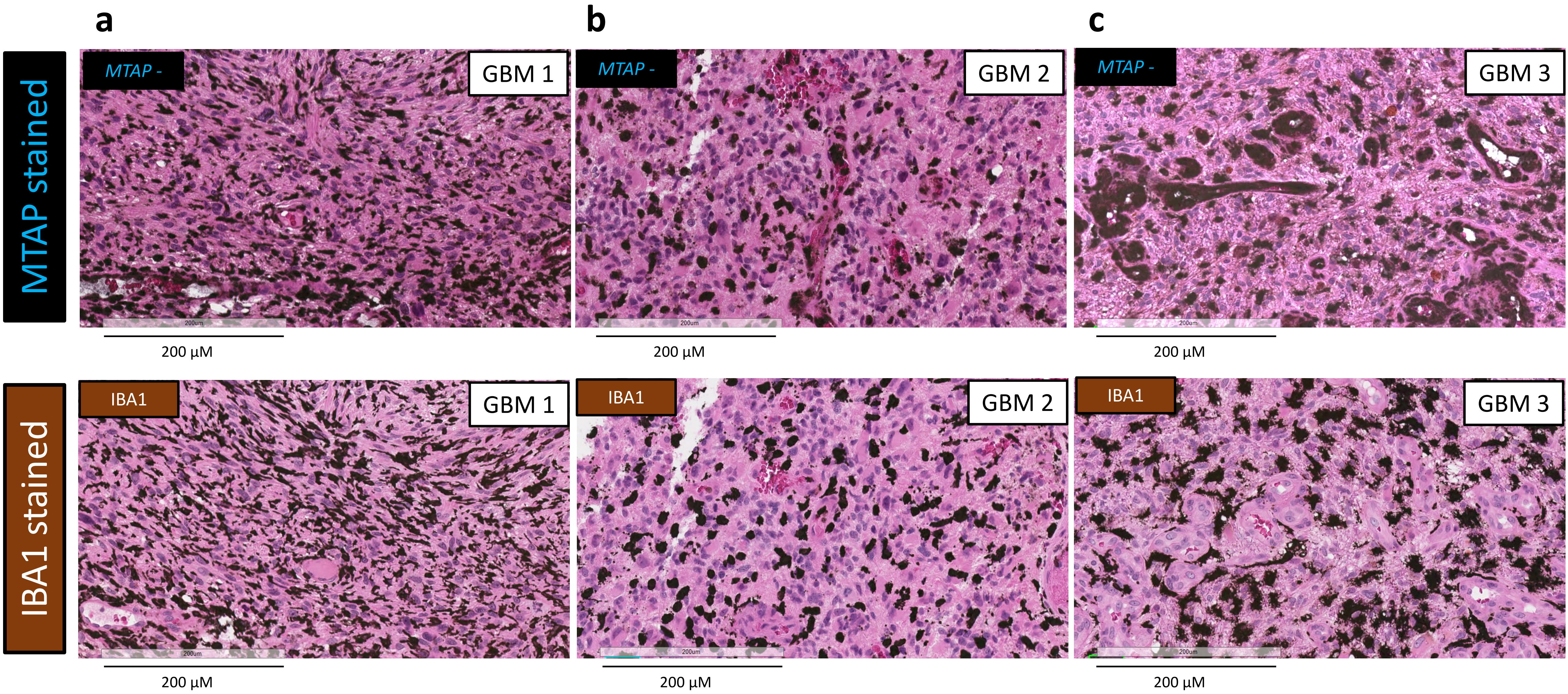

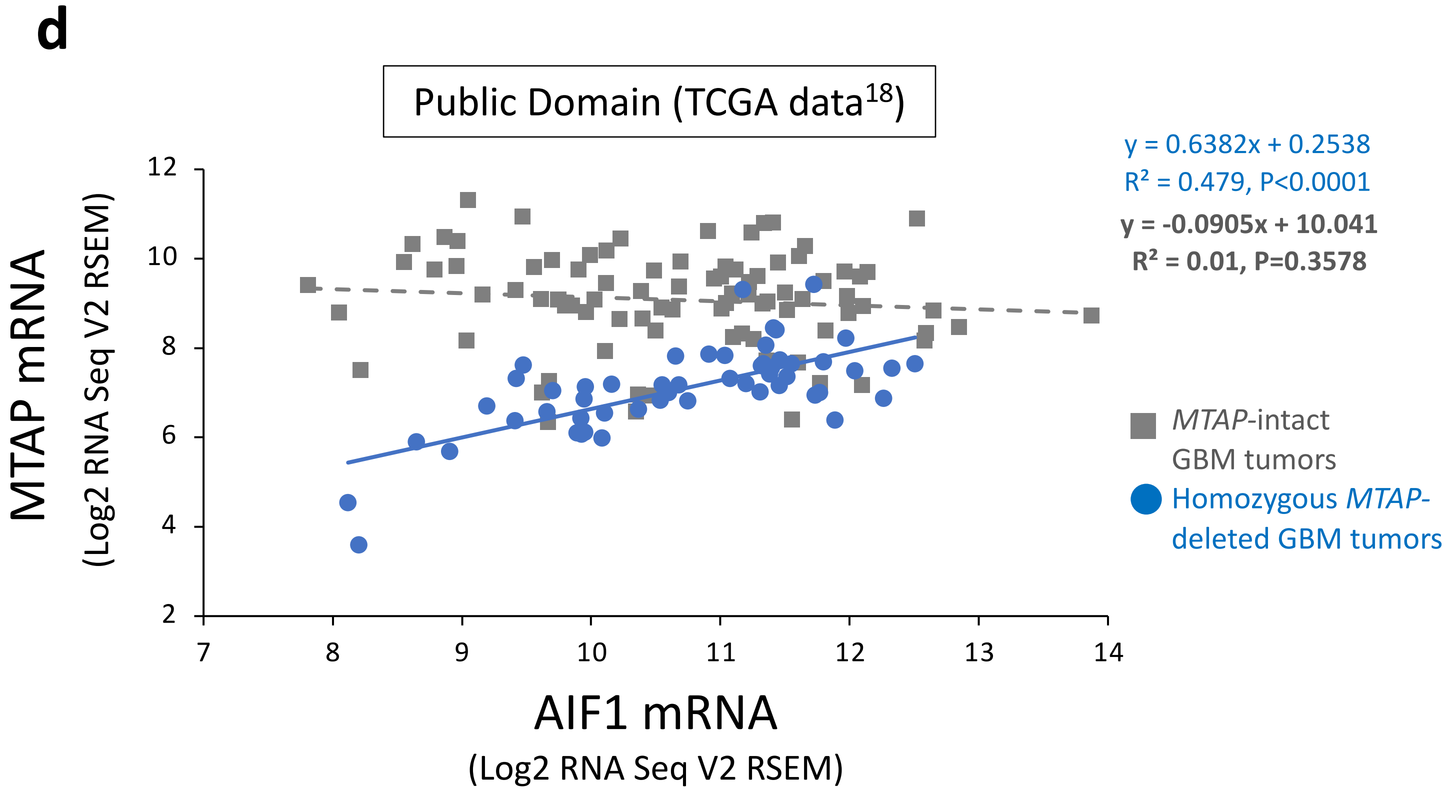

Supplementary Figure 16: Myeloid cells constitute the majority of MTAP-expressing stromal cells in MTAP-deleted GBM tumors. At least 50 FFPE serial sections were stained against MTAP (first column) and IBA1 (myeloid marker, stains macrophages/microglia, second column) and images acquired from consecutive sections of the same tumor. Three representative cases are shown here. (**a**) GBM#1, *MTAP*-deleted; (**b**) GBM#2, *MTAP*-deleted; (**c**) GBM#3, *MTAP*-deleted. Human GBM tumors may have up to 75% non-malignant stromal cell content, including *MTAP*-intact microglia. The presence of the *MTAP*-expression myeloid cells results in MTAP-positive stains even in GBM tumors that are homozygous *MTAP*-deleted since only the malignant glioma cells carry the deletion. (**d**) The correlation between MTAP mRNA levels and AIF1(microglia/macrophages marker) mRNA levels for homozygous *MTAP*-deleted (blue) and *MTAP*-intact (gray) human GBM tumors. Data obtained from TCGA Cell 2013[18] using cBioPortal[12,13]. GBM tumors are an admixture of cancer cells and non-stroma cells. Due to the presence of MTAP-expressing stroma cells such as microglia, homozygous *MTAP*-deleted GBM tumors express non-zero levels of MTAP. On average, homozygous *MTAP* deleted tumors have lower MTAP mRNA levels than *MTAP*-intact and a stronger correlation between IBA1 and MTAP for homozygous MTAP-deleted GBM tumors.

Supplementary Figure 17

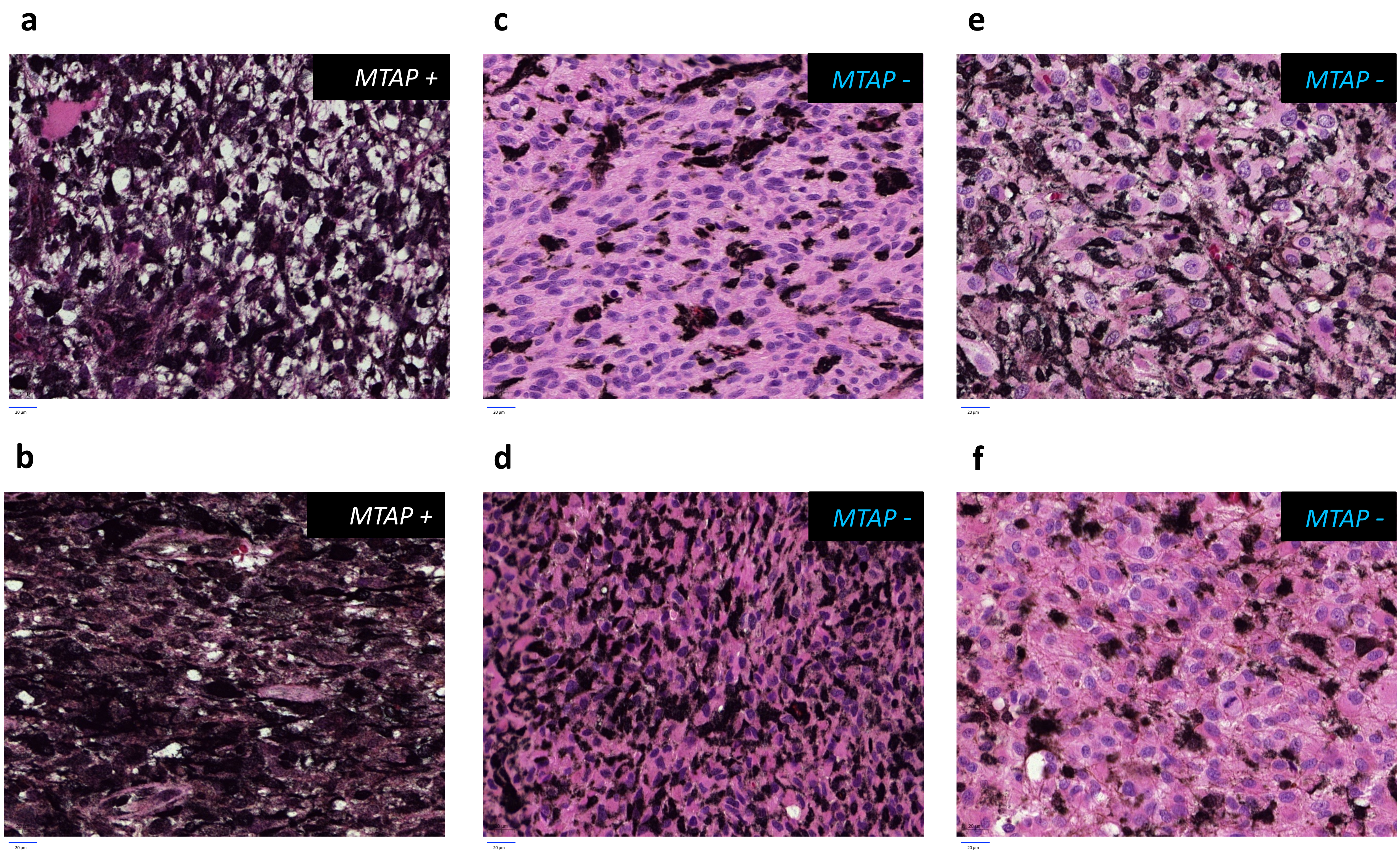

Supplementary Figure 17: Representative MTAP-deleted GBM tumors with varying stromal content. At least 50 FFPE sections were stained with anti-MTAP as in Figure 3, and images acquired using a 40X objective. Representative cases of *MTAP* positive and *MTAP* negative GBM tumors that differ in the percent of stromal content are shown. (**a, b**) *MTAP* intact – note because both stromal and tumor cells express MTAP, no distinction between stromal and tumor cells is apparent. (**c**) *MTAP*-deleted, glioma cells negative, stromal positive; (**d**) *MTAP* negative, this tumor is an example of extreme stromal content approaching 50 % cellularity. (**e**) *MTAP* negative; (**f**) *MTAP* negative; this tumor is an example of ”low” stromal content, yet note that it is still dramatically higher than in human tumors xenografted in mice (Supplementary Figures 15).

Supplementary Figure 18

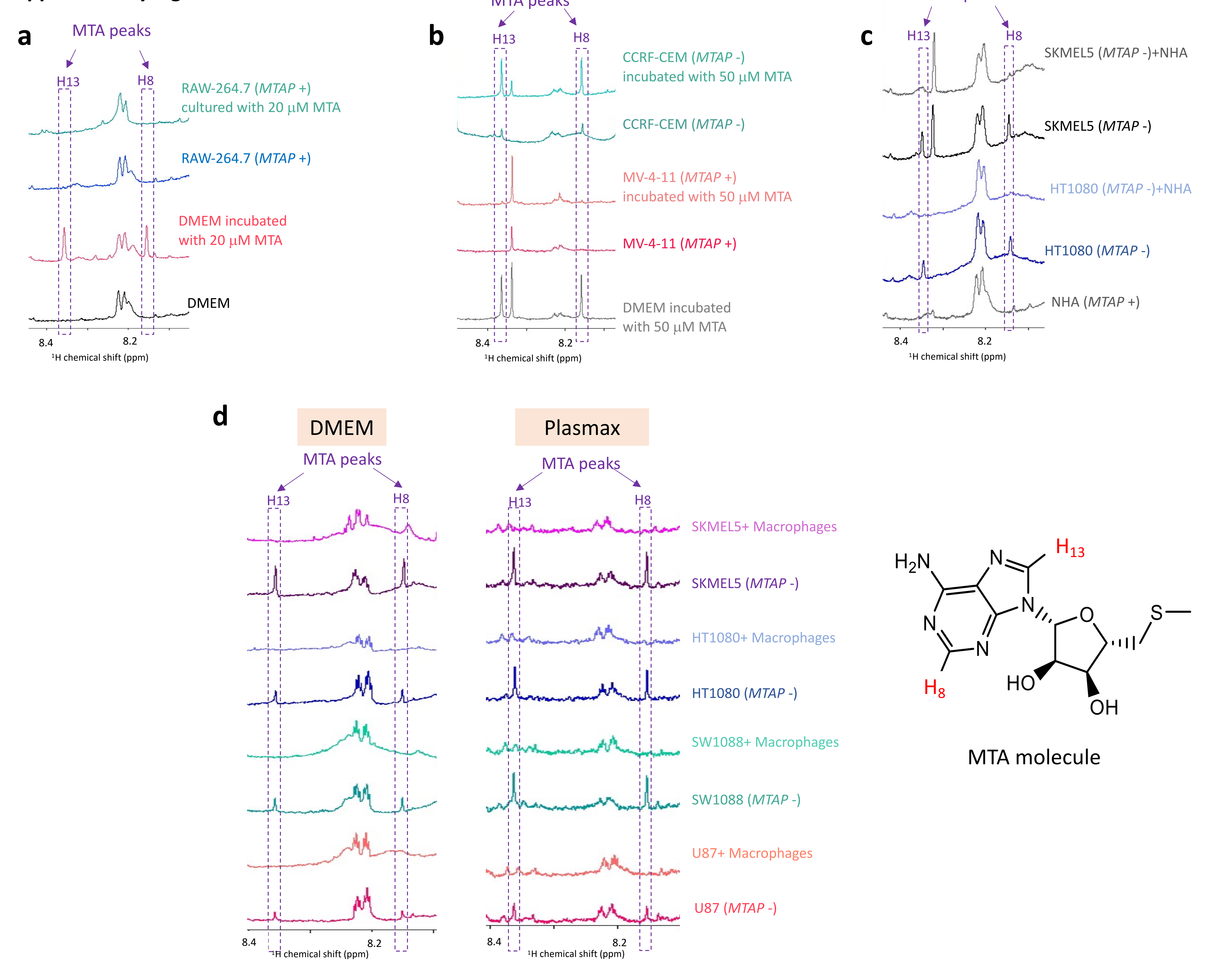

Supplementary Figure 18: Co-culture of *MTAP*-intact cells abrogates MTA accumulation in conditioned media of *MTAP*-deleted cancer cells To approximate the *in vivo* situation of stroma-admixed GBM tumors, we asked how co-culture of MTAP-expressing cells with *MTAP*-deleted cancer cells would affect levels of MTA in conditioned media. (**a**) Consumption of exogenous MTA by MTAP-expressing macrophages. Exogenous MTA was added to cultures of *MTAP*-WT macrophages (RAW-264.7) and DMEM media alone. Plates containing macrophage cells or no cells (DMEM) with and without MTA were incubated under the same experimental conditions. After 3 days, conditioned media was removed, extracted, and prepared for NMR studies. MTA peaks were detected in the spectrum of DMEM incubated with 20 μM MTA, but not in media cultured with macrophages. This indicates MTA metabolism by *MTAP*-intact macrophages cells. (**b**) Consumption of exogenous MTA by MTAP-WT but not MTAP-deleted myeloid leukemia cells. *MTAP*-WT (MV-4-11) and deleted (CCRF-CEM) myeloid leukemia cells were cultured with 50 μM of MTA in DMEM media for five days. Exogenous MTA disappeared (was consumed) from MV-4-11 (*MTAP*-WT) conditioned media, but not media in culture with CCRF-CEM (*MTAP*-deleted). (**c**) Co-culture of immortalized normal human astrocytes with *MTAP*-deleted cells prevents MTA accumulation in conditioned media. MTA was detected in conditioned media of monoculture *MTAP*-deleted cells, while it was absent from co-culture plates. In this figure, the H13 peak of MTA is overlapped with a broad peak with the same chemical shift. (**d**) Co-culture of macrophages with *MTAP*-deleted cancer cells abrogates MTA accumulation in conditioned media. Secretion by MTAP-deleted and scavenging of MTA by MTAP-WT cells are further confirmed in physiological medium (Plasmax). *MTAP*-deleted cells were cultured with and without macrophage cells (RAW-264.7) for 3 days in DMEM or Plasmax supplemented with 2.5% FBS. This figure illustrates that secreted MTA by *MTAP*-deleted cells can further metabolize by *MTAP*-intact macrophages regardless of cell culture media (DMEM vs. Plasmax), abrogating MTA accumulation in the extracellular environment.

Supplementary Figure 19

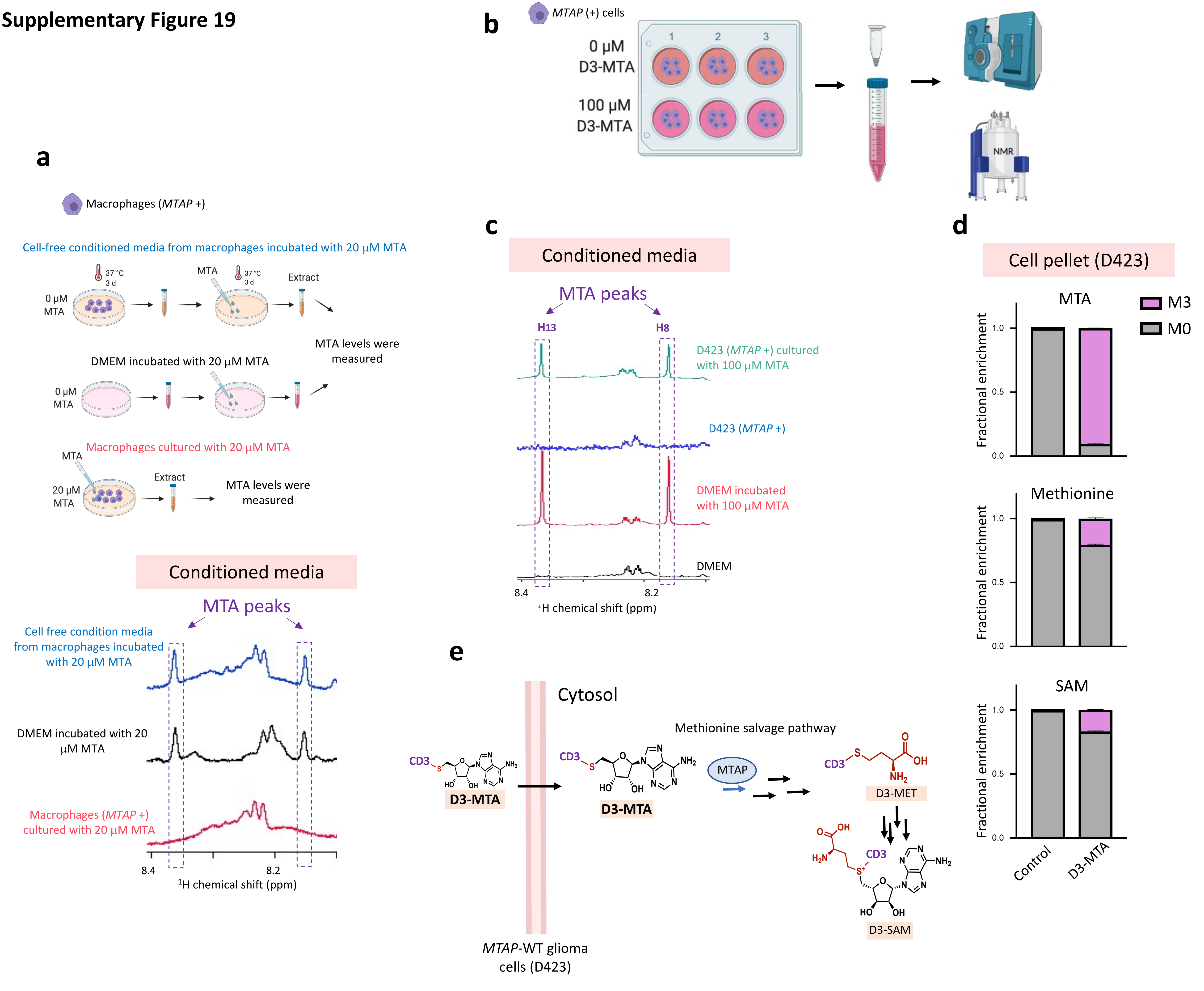

Supplementary Figure 19: Exogenous MTA is consumed by *MTAP*-intact cells through the methionine salvage pathway. (**a**) *MTAP*-intact cells (macrophages) were cultured with or without exogenous MTA (20 μM vs. 0 μM). Exogenous MTA was not eliminated from the cell-free macrophage conditioned media, while it disappeared from media cultured with macrophages. This result indicates that the release of functional MTAP enzyme by the macrophages to the extracellular environment does not contribute to exogenous MTA's elimination by *MTAP*-WT cells. Direct evidence for exogenous MTA consumption and metabolism in MTAP-intact cells. (**b**) *MTAP*-WT astroglioma cells (D423) were cultured with 100 μM tri-deuterated-MTA (D3-MTA) for 1 day. Cells and conditioned media were extracted and prepared for LC-MS and NMR measurements, respectively. (**c**) Culturing *MTAP*-WT cells with exogenous deuterated MTA results in the abrogation of D3-MTA from the conditioned media. (**d**) Intracellular enrichment of MTA, methionine, and SAM (mean +/- SD, N=3 biological replicates) after culturing *MTAP*-WT cells with the (M + 3, D3) D3-MTA label in methionine and SAM. This data indicates that MTA is taken up by *MTAP*-intact cells and metabolized further, all the way to methionine through the methionine salvage pathway. (**e**) The fate of deuterium from exogenous labeled D3-MTA into methionine (methionine salvage pathway) and SAM (polyamine biosynthesis).

Supplementary Figure 20

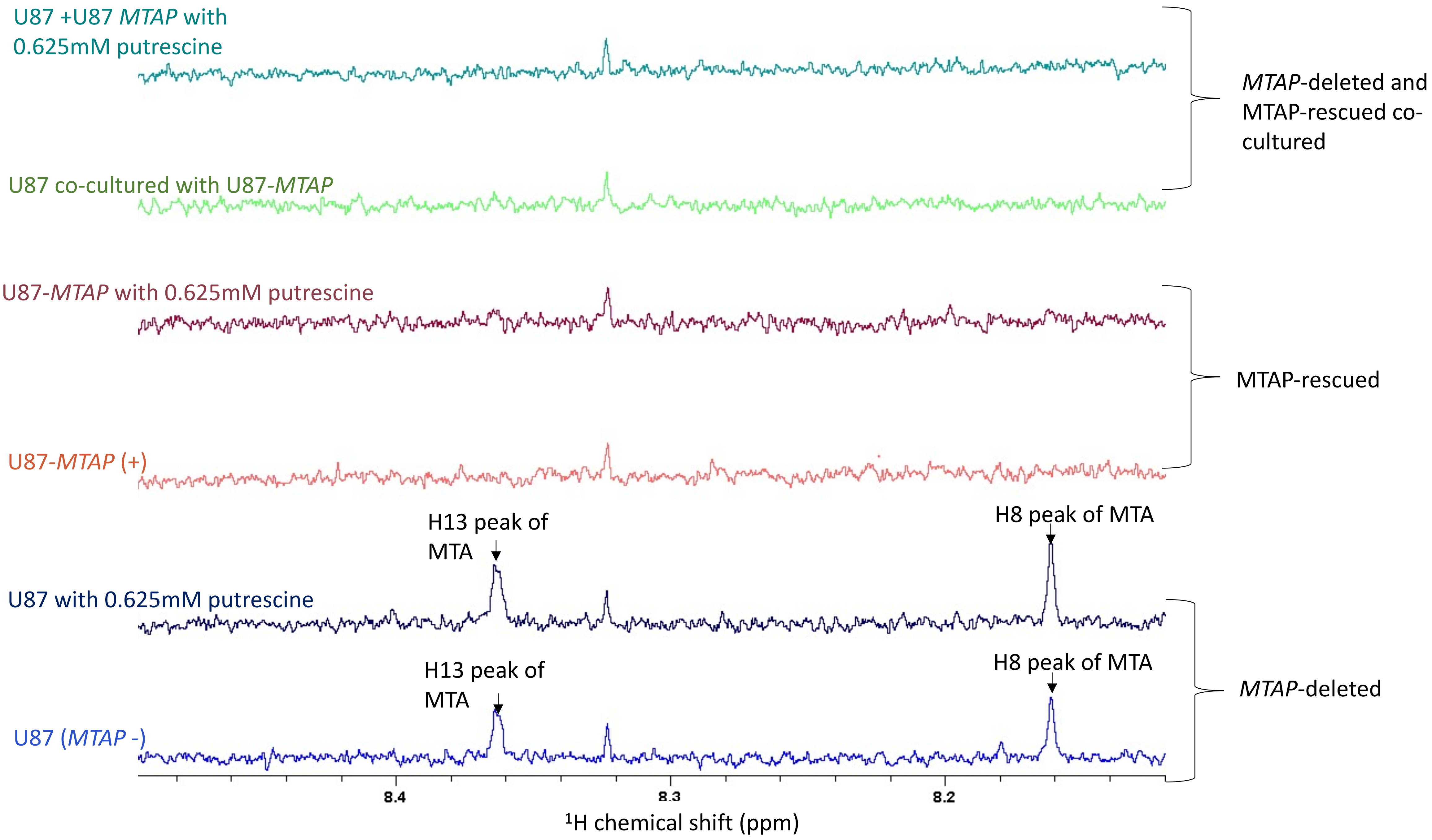

Supplementary Figure 20: The co-culture of *MTAP*-deleted and *MTAP*-rescued glioma cells abrogates MTA accumulation from conditioned media. Equal numbers of U87 and U87 *MTAP*-rescued cells were co-cultured for 3 days with or without treatment with 0.625 mM putrescine (to stimulate MTA formation, Kamatani, and Carson, Cancer Res. 1980[19]). After 3 days, media was extracted with ethyl acetate and prepared for NMR studies. The two peaks of H13 and H8 of MTA appear in the U87 and U87 treated with putrescine media extract, while they are absent in U87-*MTAP* and U87+U87-*MTAP* media extract. This figure shows putrescine increases the amount of MTA in the media.

Supplementary References

1. Marjon, K. *et al.* MTAP Deletions in Cancer Create Vulnerability to Targeting of the MAT2A/PRMT5/RIOK1 Axis. *Cell Rep.* **15**, 574–587 (2016).
2. Mavrakis, K. J. *et al.* Disordered methionine metabolism in MTAP/CDKN2A-deleted cancers leads to dependence on PRMT5. *Science (80-.).* **351**, 1208–1213 (2016).
3. Hörmann, A. *et al.* RIOK1 kinase activity is required for cell survival irrespective of MTAP status. *Oncotarget* **9**, (2018).
4. Gao, G. *et al.* PRMT1 loss sensitizes cells to PRMT5 inhibition. *Nucleic Acids Res.* **47**, 5038–5048 (2019).
5. Fedoriw, A. *et al.* Anti-tumor Activity of the Type I PRMT Inhibitor, GSK3368715, Synergizes with PRMT5 Inhibition through MTAP Loss. *Cancer Cell* **36**, 100-114.e25 (2019).
6. Ortmayr, K., Dubuis, S. & Zampieri, M. Metabolic profiling of cancer cells reveals genome-wide crosstalk between transcriptional regulators and metabolism. *Nat. Commun.* **10**, 1841 (2019).
7. Dettmer, K. *et al.* Distinct metabolic differences between various human cancer and primary cells. *Electrophoresis* **34**, n/a-n/a (2013).
8. Su, G., Burant, C. F., Beecher, C. W., Athey, B. D. & Meng, F. Integrated metabolome and transcriptome analysis of the NCI60 dataset. *BMC Bioinformatics* **12 Suppl 1**, S36 (2011).
9. Kryukov, G. V *et al.* MTAP deletion confers enhanced dependency on the PRMT5 arginine methyltransferase in cancer cells. *Science (80-.).* **351**, 1214–1218 (2016).
10. Prabhu, A. H. *et al.* Integrative cross-platform analyses identify enhanced heterotrophy as a metabolic hallmark in glioblastoma. *Neuro. Oncol.* **21**, 337–347 (2019).
11. http://gdac.broadinstitute.org/runs/stddata__2016_01_28/data/GBM/20160128/
12. Gao, J. *et al.* Integrative analysis of complex cancer genomics and clinical profiles using the cBioPortal. *Sci. Signal.* **6**, 1–20 (2013).
13. Cerami, E. *et al.* The cBio Cancer Genomics Portal: An open platform for exploring multidimensional cancer genomics data. *Cancer Discov.* **2**, 401–404 (2012).
14. Chinnaiyan, P. *et al.* Molecular and Cellular Pathobiology The Metabolomic Signature of Malignant Glioma Reflects Accelerated Anabolic Metabolism. *Cancer Res.* **72**, 5878–88. (2012).
15. Sanderson, S. M., Mikhael, P. G., Ramesh, V., Dai, Z. & Locasale, J. W. Nutrient availability shapes methionine metabolism in p16/ *MTAP* -deleted cells. *Sci. Adv.* **5**, eaav7769 (2019).
16. Locasale, J. W. *et al.* Metabolomics of Human Cerebrospinal Fluid Identifies Signatures of Malignant Glioma. *Mol. Cell. Proteomics* **11**, M111.014688 (2012).
17. Xiong, N. *et al.* Using arterial–venous analysis to characterize cancer metabolic consumption in patients. *Nat. Commun.* **11**, (2020).
18. Brennan, C. W. *et al.* The Somatic Genomic Landscape of Glioblastoma. *Cell* **155**, 462–477 (2013).
19. Kamatani, N. & Carson, D. A. Abnormal Regulation of Methylthioadenosine and Polyamine Metabolism in Methylthioadenosine Phosphorylase-deficient Human Leukemic Cell Lines. *Cancer Res.* **40**, 4178–4182 (1980).
