# Supplementary material for: Homozygous MTAP deletion in primary human glioblastoma is not associated with elevation of methylthioadenosine
Source: Nat Commun. 2021 Jul 9;12:4228. doi: 10.1038/s41467-021-24240-3 (PMC8270912; doi:10.1038/s41467-021-24240-3)
Supplement: Supplementary file 3 — Reporting summary [file 41467_2021_24240_MOESM3_ESM.pdf]

nature research

Corresponding author(s): Florian Muller

Last updated by author(s): May 22, 2021

Reporting Summary

Nature Research wishes to improve the reproducibility of the work that we publish. This form provides structure for consistency and transparency in reporting. For further information on Nature Research policies, see our Editorial Policies and the Editorial Policy Checklist.

Statistics

For all statistical analyses, confirm that the following items are present in the figure legend, table legend, main text, or Methods section.

n/a	Confirmed	
☐	☒	The exact sample size (*n*) for each experimental group/condition, given as a discrete number and unit of measurement
☐	☒	A statement on whether measurements were taken from distinct samples or whether the same sample was measured repeatedly
☐	☒	The statistical test(s) used AND whether they are one- or two-sided<br>*Only common tests should be described solely by name; describe more complex techniques in the Methods section.*
☒	☐	A description of all covariates tested
☐	☒	A description of any assumptions or corrections, such as tests of normality and adjustment for multiple comparisons
☐	☒	A full description of the statistical parameters including central tendency (e.g. means) or other basic estimates (e.g. regression coefficient) AND variation (e.g. standard deviation) or associated estimates of uncertainty (e.g. confidence intervals)
☐	☒	For null hypothesis testing, the test statistic (e.g. *F*, *t*, *r*) with confidence intervals, effect sizes, degrees of freedom and *P* value noted<br>*Give P values as exact values whenever suitable.*
☒	☐	For Bayesian analysis, information on the choice of priors and Markov chain Monte Carlo settings
☒	☐	For hierarchical and complex designs, identification of the appropriate level for tests and full reporting of outcomes
☒	☐	Estimates of effect sizes (e.g. Cohen's *d*, Pearson's *r*), indicating how they were calculated

Our web collection on statistics for biologists contains articles on many of the points above.

Software and code

Policy information about availability of computer code

Data collection	NMR spectra were acquired on a Bruker NMR and were analyzed with Bruker's Topspin 3.1 software. This is a standard in the field and should prove no barrier for other researchers to review our data.
Data analysis	NMR data analysis was performed with Topspin, which is both commercially available from Bruker (Berilica, MA). Isotope labeling data were analyzed using El-Maven 7.0 software (Elucidata, LLC., elucidata.io.). Targeted mass spectroscopy data were analyzed using MultiQuant v2.1 software. Western blot bands were quantified using ImageJ 1.52q.

For manuscripts utilizing custom algorithms or software that are central to the research but not yet described in published literature, software must be made available to editors and reviewers. We strongly encourage code deposition in a community repository (e.g. GitHub). See the Nature Research guidelines for submitting code & software for further information.

Data

Policy information about availability of data

All manuscripts must include a data availability statement. This statement should provide the following information, where applicable:

- Accession codes, unique identifiers, or web links for publicly available datasets
- A list of figures that have associated raw data
- A description of any restrictions on data availability

Metabolamics data and uncropped scans of all blots are provided in the Source Data file. All metabolamics and NMR data are deposited in Figshare (https://doi.org/10.6084/m9.figshare.14608002.v1). Data in Supplementary Figures S6G and S16D are available in cBioPortal (https://www.cbioportal.org). Public domain metabolamics data used in Supplementary Figures S2a, S2b, S2c, S4a, S6, S7, S9, and S11 are available at https://doi.org/10.1038/s41467-019-09695-9, 10.1186/1471-2105-12-S1-S36, 10.1002/elps.201300228, 10.1126/science.aad5214, 10.1093/neuonc/noy185, 10.1158/0008-5472.CAN-12-1572-T, 10.1126/sciadv.aav7769, and 10.1038/s41467-020-16810-8 respectively. All other data supporting the findings of this study are available from the article and the

supplementary information files and from the corresponding author upon reasonable request. Source data are provided with this paper.

Field-specific reporting

Please select the one below that is the best fit for your research. If you are not sure, read the appropriate sections before making your selection.

[x] Life sciences   [ ] Behavioural & social sciences   [ ] Ecological, evolutionary & environmental sciences

For a reference copy of the document with all sections, see nature.com/documents/nr-reporting-summary-flat.pdf

Life sciences study design

All studies must disclose on these points even when the disclosure is negative.

Sample size	Sample size was chosen based on the common standards in the cancer literature. Sample size of the number of independently interrogated biological samples was limited by the number of cell lines with the desired genotype available. Three biological replicates were used for cell culture experiments. The number of primary GBM tumors were limited by the difficulty to obtain such tumors. The number of biological replicates for primary GBM tumors were also limited by the amount. We performed metabolomics on 40 primary GBM tumors (biollagically independent samples).
Data exclusions	Except for gross technical mistakes (broken NMR tubes, spilled or unloaded samples, low sample loading and contamination) no data were excluded
Replication	We confirmed secretion of MTA by MTAP-deleted cells in our panel of cell lines using two independent methods (NMR and mass-spect) as well as public domain data (Supplimentary Figure S2C). MTA secretion and excavation by myloid cells are also confirmed in super-physiological and physiological media using two different methods (NMR and mass-spect). All attempts at replication were successful. The conclusion of no significant MTA accumulation in primary GBM tumors was reached by analyzing of two independent different dataset (one from HF series using BIDMC and another one MDA series using Metabolon, Inc. We further test our conclusions by interrogating independent GBM metabolomic datasets in the literature.
Randomization	No randomization was performed as this would not be practical. For this study, we were comparing MTA levels in MTAP-deleted vs. WT, to test the hypothesis of whether MTA levels is elevated in MTAP-deleted primary GBM tumors or not. Thus, the randomization was not possible and necessary.
Blinding	No systematic and deliberate blinding of the experimentalist was performed; however, the experimentalist was not typically aware of what the genotype of a given sample was, thus

Reporting for specific materials, systems and methods

We require information from authors about some types of materials, experimental systems and methods used in many studies. Here, indicate whether each material, system or method listed is relevant to your study. If you are not sure if a list item applies to your research, read the appropriate section before selecting a response.

Materials & experimental systems

n/a	Involved in the study	
[ ]	[x]	Antibodies
[ ]	[x]	Eukaryotic cell lines
[x]	[ ]	Palaeontology and archaeology
[ ]	[x]	Animals and other organisms
[ ]	[x]	Human research participants
[x]	[ ]	Clinical data
[x]	[ ]	Dual use research of concern

Methods

n/a	Involved in the study	
[x]	[ ]	ChIP-seq
[x]	[ ]	Flow cytometry
[x]	[ ]	MRI-based neuroimaging

Antibodies

Antibodies used	A detailed description of all the antibodies are provided in the method section.<br>The following antibodies are used for this study: Symmetricdi-methylargininemotif (Cell SignalingTechnology, #13222, Lot: 6) in a 1:1000 dilution for western blot, MTAP (Abcamab126623, Lot: GR97816-5, Lot: YI070108C5, Lot: GR90092-13) in a 1:1000 for western blot and 1:250 for IHC , GAPDH (Sigma, G9545, lot: 127M4814V) in a 1:5000 dilution for western blot, Vinculin (Cell Signaling Technology, #13901) in a 1:5000 dilution for western blot, and IBA1 (Abcam, #ab178846, lot: GR207976-27) in a 1:1000 dilution for both IHC and western blot. 1× goat anti-rabbit IgG secondary antibody, poly–horseradish peroxidase conjugate (Invitrogen by Thermo Scientific, Ref:B40962, Lot: 2140280).
Validation	MTAP antibody was validated a monoclonal antibody for this application by demonstrating staining in FFPE slides of

xenografted tumors with known MTAP genotype. SDMA antibody is also validated by performing western blot on MTAP-deleted cells treated with/without MTA and MTAP-WT cells. The levels of SDMA measured using this antibody was less in MTAP-deleted cells than MTAP-WT and decreased more followed by treatment with MTA, as expected based on the literature. Other antibodies (e.i., Vinculin, GAPDH, IBA1) are the standard in the field and have been extensively validated by the manufactures (Cell Signaling, Abcam, Sigma).

Eukaryotic cell lines

Policy information about cell lines

Cell line source(s)	The cell lines used in this study that are MTAP-WT were D423 (CVCL_1160, H423/D423-MG) and D502 (CVCL_1162, H502) were kindly provided by Darrel Bigner58. U343 (CVCL_S471, U343-MG), LN319 (CVCL_3958, a sub-clone of LN-992), and NB1 (CVCL_1440) were obtained from the Department of Genomic Medicine/Institute for Applied Cancer Science Cell Bank at MD Anderson. MV-4-11 (CVCL_0064) purchased from NCI, RAW-264.7 (CVCL_0493) purchased from ATCC (TIB-71), and immortalized normal human astrocytes was kindly provided by Dr. Seth Gammon (Dept of Cancer System Imaging). The MTAP-deleted cell lines were U87 (CVCL-0022), SW1088 (CVCL_1715), and SKMEL5 (CVCL_0527) obtained from the Department of Genomic Medicine/Institute for Applied Cancer Science Cell Bank at MD Anderson. Gli56 (D. Louis), CCRF-CEM (CVCL_0207) purchased from NCI. HT1080 (CVCL_0317) was kindly provided by Dr. Seth Gammon (Cancer Systems Imaging).
Authentication	Cell lines were authenticated by STR testing at MD Anderson's Characterized Cell Line Core
Mycoplasma contamination	Cell lines were routinely tested for mycoplasma contamination using a commercial ELISA kit, and all were tested negative for mycoplasma contamination
Commonly misidentified lines (See ICLAC register)	U-87, obtained from ATCC. Issues around mismatch between U87-ATCC and the original donor have been described; however, this is not an issue for interpretation, as U87-ATCC is confirmed as MTAP-deleted.

Animals and other organisms

Policy information about studies involving animals; ARRIVE guidelines recommended for reporting animal research

Laboratory animals	Xenografted tumors in immunocompromised Foxn1 nude mice were employed between the ages of 7-25 weeks, female mice from M.D. Anderson's Department of Experimental Radiation Oncology (ERO).
Wild animals	No wild animals were used in the study.
Field-collected samples	No field-collected samples were used in the study.
Ethics oversight	All procedures were approved by M.D. Anderson's Institutional Care and Use Committee (IACUC)

Note that full information on the approval of the study protocol must also be provided in the manuscript.

Human research participants

Policy information about studies involving human research participants

Population characteristics	De-identified, archival GBM tumors were studied as part of the manuscript. As a result of de-identification, no demographic information is available.
Recruitment	GBM were consented and collected during resection surgeries under the approved institutional review board (IRB) protocol by the MD Anderson(PA15-0940). No self-selection bias or other biases
Ethics oversight	

Note that full information on the approval of the study protocol must also be provided in the manuscript.
